# Supplementary material for: A Pseudomonas Lysogenic Bacteriophage Crossing the Antarctic and Arctic, Representing a New Genus of Autographiviridae
Source: Int J Mol Sci. 2023 Apr 21;24(8):7662. doi: 10.3390/ijms24087662 (PMC10142737; doi:10.3390/ijms24087662)
Supplement: Supplementary file 1 [file ijms-24-07662-s001.zip › ijms-2334713-supplementary.pdf]

Table S1. Identified prophage regions of *Pseudomonas* sp. D3.

Region	Region Length	Completeness	Score	# Total Proteins	Most Common Phage	GC %
1	65.1Kb	intact	150	83	PHAGE_Vibrio_VP882_NC_009016(15)	58.74%
2	26.9Kb	incomplete	50	15	PHAGE_Burkho_phi1026b_NC_005284(2)	57.51%
3	42.8Kb	questionable	82	49	PHAGE_Pseudo_Dobby_NC_048109(27)	57.39%
4	44.1Kb	intact	110	33	PHAGE_Pseudo_YMC11/02/R656_NC_028657(12)	59.21%
5	23.2Kb	questionable	90	17	PHAGE_Pseudo_YMC11/02/R656_NC_028657(3)	56.26%
6	40.8Kb	incomplete	40	37	PHAGE_Pseudo_phiPsa17_NC_047747(15)	54.01%
7	6.7Kb	incomplete	40	7	PHAGE_Salmon_SJ46_NC_031129(3)	51.71%
8	41.8Kb	questionable	70	54	PHAGE_Pseudo_MD8_NC_031091(11)	58.32%
9	44Kb	intact	150	53	PHAGE_Pseudo_F10_NC_007805(14)	58.35%
10	30.1Kb	incomplete	40	15	PHAGE_Escher_500465_1_NC_049342(5)	58.95%
11	22.2Kb	incomplete	30	6	PHAGE_Klebsi_ST147_VIM1phi7.1_NC_049451(2)	59.47%

Total: 11 prophage regions have been identified, of which 3 regions are intact, 5 regions are incomplete, and 3 regions are questionable.

Intact (score > 90)

Questionable (score 70-90)

Incomplete (score < 70)

Region: The number assigned to the region.

Region Length: The length of the sequence of that region (in bp).

Completeness: A prediction of whether the region contains an intact or incomplete prophage based on the above criteria.

Score: The score of the region is based on the above criteria.

# Total Proteins: The number of ORFs present in the region.

Most Common Phage: The phage(s) with the highest number of proteins most similar to those in the region.

GC %: The percentage of GC nucleotides of the region.

Table S2. Pairwise comparisons of *Pseudomonas* sp. D3 and *Pseudomonas* sp. G11 genomes vs. type strain genomes.

Query strain	Subject strain	dDDH (d0, in %)	dDDH (d4, in %)	dDDH (d6, in %)
Pseudomonas sp. D3	*Pseudomonas veronii* DSM 11331	67.2	54.4	66.3
Pseudomonas sp. G11	*Pseudomonas grimontii* DSM 17515	55.8	37.4	51.5
Pseudomonas sp. G11	*Pseudomonas marginalis* DSM 13124	55.1	37.2	50.9
Pseudomonas sp. D3	*Pseudomonas fildesensis* KG01	58.1	37	53.2
Pseudomonas sp. D3	*Pseudomonas* sp. G11	51.8	36.8	48.1
Pseudomonas sp. D3	*Pseudomonas extremaustralis* 14-3	55	36.6	50.6
Pseudomonas sp. G11	*Pseudomonas fildesensis* KG01	54.2	36.5	49.9
Pseudomonas sp. G11	*Pseudomonas veronii* DSM 11331	55.2	36.2	50.6
Pseudomonas sp. D3	*Pseudomonas grimontii* DSM 17515	52.3	35.5	48
Pseudomonas sp. G11	*Pseudomonas aylmerensis* S1E40	49.5	35.5	45.9
Pseudomonas sp. G11	*Pseudomonas extremaustralis* 14-3	48.2	35.3	44.8
Pseudomonas sp. D3	*Pseudomonas marginalis* DSM 13124	51.3	35.3	47.2
Pseudomonas sp. D3	*Pseudomonas aylmerensis* S1E40	49.4	34.9	45.6
Pseudomonas sp. G11	*Pseudomonas cyclaminis* MAFF 301449T	48.5	34.8	44.9
Pseudomonas sp. G11	*Pseudomonas petroselini* MAFF 311094	51.9	34.7	47.5
Pseudomonas sp. G11	*Pseudomonas pergaminensis* 1008T	51	34.6	46.7
Pseudomonas sp. G11	*Pseudomonas allii* MAFF 301514	53.4	34.6	48.6
Pseudomonas sp. G11	*Pseudomonas azotoformans* LMG 21611	51.8	34.6	47.3
Pseudomonas sp. G11	*Pseudomonas marginalis* ICMP 3553	52.1	34.5	47.6
Pseudomonas sp. D3	*Pseudomonas petroselini* MAFF 311094	48.6	34.5	44.9
Pseudomonas sp. D3	*Pseudomonas azotoformans* LMG 21611	50.3	34.4	46.2
Pseudomonas sp. G11	*Pseudomonas extremorientalis* LMG 19695	52.5	34.4	47.8
Pseudomonas sp. D3	*Pseudomonas marginalis* ICMP 3553	50.8	34.4	46.5
Pseudomonas sp. D3	*Pseudomonas pergaminensis* 1008T	50.1	34.3	46
Pseudomonas sp. D3	*Pseudomonas extremorientalis* LMG 19695	51.3	34.2	46.8
Pseudomonas sp. D3	*Pseudomonas cyclaminis* MAFF 301449T	48.9	34.2	45
Pseudomonas sp. D3	*Pseudomonas allii* MAFF 301514	49.4	34.2	45.4
Pseudomonas sp. G11	*Pseudomonas antarctica* LMG 22709	45.5	33.8	42.2
Pseudomonas sp. G11	*Pseudomonas asgharzadehiana* SWRI132	52	33.8	47.2
Pseudomonas sp. G11	*Pseudomonas sivasensis* P7	49.1	33.8	45
Pseudomonas sp. G11	*Pseudomonas simiae* CCUG 50988	52.6	33.7	47.6
Pseudomonas sp. D3	*Pseudomonas simiae* CCUG 50988	53.8	33.6	48.5
Pseudomonas sp. D3	*Pseudomonas asgharzadehiana* SWRI132	53	33.6	47.9
Pseudomonas sp. D3	*Pseudomonas antarctica* LMG 22709	50.3	33.6	45.8
Pseudomonas sp. D3	*Pseudomonas sivasensis* P7	50.2	33.3	45.6
Pseudomonas sp. D3	*Pseudomonas kitaguniensis* MAFF 212408T	43.3	32.5	40
Pseudomonas sp. G11	*Pseudomonas kitaguniensis* MAFF 212408T	39.3	32.4	36.9

Table S3. The source of 95 strains of *Pseudomonas*.

CCTCC No.	Name*	Isolated_from	Country
CCTCC S2012104	*Pseudomonas baetica*	Soil	Arctic Ny-Alesund
CCTCC S2012113	*Pseudomonas reinekei*	Soil	Arctic Ny-Alesund
CCTCC S2012311	*Pseudomonas koreensis*	Soil	Arctic Ny-Alesund
CCTCC S2012564	*Pseudomonas punonensis*	Soil	Arctic
CCTCC S2012668	*Pseudomonas pelagia*	Soil	Arctic Ny-Alesund
CCTCC S2013037	*Pseudomonas cedrina*	Soil	Fildes Peninsula, Antarctica
CCTCC S2013231	*Pseudomonas brenneri*	Soil	Arctic Ny-Alesund
CCTCC S2013262	*Pseudomonas extremaustralis*	Soil	Arctic Ny-Alesund
CCTCC S2013472	*Pseudomonas psychrotolerans*	Feces	Fildes Peninsula, Antarctica
CCTCC S2013652	*Pseudomonas cedrina* subsp.	Soil	Fildes Peninsula, Antarctica
CCTCC S2013668	*Pseudomonas flavescens*	Soil	Antarctica
CCTCC S2013684	*Pseudomonas taeanensis*	Soil	Antarctica
CCTCC S2014069	*Pseudomonas thivervalensis*	Soil	Fildes Peninsula, Antarctica
CCTCC S2014099	*Pseudomonas brenneri*	Soil	Fildes Peninsula, Antarctica
CCTCC S2014108	*Pseudomonas antarctica*	Soil	Fildes Peninsula, Antarctica
CCTCC S2014299	*Pseudomonas syringae*	Soil	Fildes Peninsula, Antarctica
CCTCC S2014340	*Pseudomonas brenneri*	Soil	Fildes Peninsula, Antarctica
CCTCC S2014342	*Pseudomonas poae*	Soil	Fildes Peninsula, Antarctica
CCTCC S2014365	*Pseudomonas baetica*	Soil	Fildes Peninsula, Antarctica
CCTCC S2014426	*Pseudomonas syringae*	Soil	Fildes Peninsula, Antarctica
CCTCC S2014584	*Pseudomonas avellanae*	Soil	Arctic Ny-Alesund
CCTCC S2014754	*Pseudomonas meridiana*	Soil	Arctic Ny-Alesund
CCTCC S2014863	*Pseudomonas azotoformans*	Soil	Fildes Peninsula, Antarctica
CCTCC S2014901	*Pseudomonas protegens*	Flowers	Arctic Ny-Alesund
CCTCC S2014926	*Pseudomonas avellanae*	Flowers	Arctic Ny-Alesund
CCTCC S2015032	*Pseudomonas graminis*	Soil	Arctic Longyear
CCTCC S2015124	*Pseudomonas chlororaphis subsp.piscium*	Soil	Arctic Longyear
CCTCC S2015145	*Pseudomonas chlororaphis subsp.piscium*	Soil	Arctic Ny-Alesund
CCTCC AB2015149	*Pseudomonas piscium*	Soil	South Shetland Islands, Antarctica
CCTCC S2015157	*Pseudomonas ficuserectae*	Soil	Arctic Ny-Alesund
CCTCC S2015203	*Pseudomonas rhizosphaerae*	Flowers	Arctic Ny-Alesund
CCTCC S2015248	*Pseudomonas gessardii*	Feces	Antarctica Fildes Peninsula
CCTCC S2015249	*Pseudomonas guineae*	Feces	Antarctica Fildes Peninsula
CCTCC S2015252	*Pseudomonas caeni*	Feces	Antarctica Fildes Peninsula
CCTCC S2015320	*Pseudomonas salomonii*	Soil	South Shetland Islands, Antarctica
CCTCC S2015388	*Pseudomonas brenneri*	Soil	Arctic Longyear
CCTCC S2015407	*Pseudomonas frederiksbergensis*	Soil	Arctic Longyear
CCTCC S2015434	*Pseudomonas beteli*	Reindeer droppings	Arctic Ny-Alesund
CCTCC S2015441	*Pseudomonas beteli*	Reindeer droppings	Arctic Ny-Alesund
CCTCC S2015639	*Pseudomonas meridiana*	Soil	South Shetland Islands, Antarctica
CCTCC S2015652	*Pseudomonas libanensis*	Soil	Arctic Ny-Alesund
CCTCC S2015847	*Pseudomonas libanensis*	Soil	Antarctic
CCTCC AB 2016039	*Pseudomonas fragi*	Soil	Arctic
CCTCC S2016218	*Pseudomonas chlororaphis*	Moraine	Arctic Ny-Alesund
CCTCC S2016494	*Pseudomonas cryoconiti*	Soil	Antarctica
CCTCC S2016531	*Pseudomonas chlororaphis subsp. piscium*	Soil	Antarctica

CCTCC S2016797	*Pseudomonas marginalis*	Soil	Antarctica
CCTCC S2016804	*Pseudomonas koreensis*	Soil	Antarctica
CCTCC S2017030	*Pseudomonas arsenicoxydans*	Soil	Antarctic Penguin Island
CCTCC S2017091	*Pseudomonas ficuserectae*	Soil	Fildes Peninsula, Antarctica
CCTCC S2017137	*Pseudomonas migulae*	Soil	Fildes Peninsula, Antarctica
CCTCC S2017165	*Pseudomonas frederiksbergensis*	Soil	Fildes Peninsula, Antarctica
CCTCC S2017205	*Pseudomonas taetrolens*	Soil	Fildes Peninsula, Antarctica
CCTCC S2017300	*Pseudomonas yamanorum*	Soil	Fildes Peninsula, Antarctica
CCTCC S2017306	*Pseudomonas weihenstephanensis*	Soil	Fildes Peninsula, Antarctica
CCTCC S2017310	*Pseudomonas simiae*	Soil	Fildes Peninsula, Antarctica
CCTCC AB 2018326	*Pseudomonas simiae*	Reindeer droppings	Arctic Ny-Alesund
CCTCC AB 2018332	*Pseudomonas* sp. D3	Soil	Fildes Peninsula, Antarctica
CCTCC AB 2018339	*Pseudomonas guineae*	Marine sediments	Arctic Longyear
CCTCC AB 2018340	*Pseudomonas fluorescens*	Soil	Fildes Peninsula, Antarctica
CCTCC AB 2018344	*Pseudomonas baetica*	Marine sediments	Arctic Longyear
CCTCC S2019025	*Pseudomonas coronafaciens*	Soil	Fildes Peninsula, Antarctica
CCTCC S2019058	*Pseudomonas frederiksbergensis*	Soil	Fildes Peninsula, Antarctica
CCTCC S2019077	*Pseudomonas mandelii*	Soil	Fildes Peninsula, Antarctica
CCTCC S2019263	*Pseudomonas arsenicoxydans*	Soil	Norway
CCTCC S2019297	*Pseudomonas frederiksbergensis*	Soil	Arctic
CCTCC S2019309	*Pseudomonas silesiensis*	Soil	Arctic
CCTCC S2019310	*Pseudomonas prosekii*	Soil	Arctic
CCTCC S2019316	*Pseudomonas avellanae*	Soil	Arctic
CCTCC S2019358	*Pseudomonas guineae*	Glacier	Arctic Yellow River Station
CCTCC S2019381	*Pseudomonas grimontii*	Nectar	Arctic Yellow River Station
CCTCC S2019393	*Pseudomonas extremaustralis*	Nectar	Arctic Yellow River Station
CCTCC S2019471	*Pseudomonas proseki*	Glacier	Arctic Yellow River Station
CCTCC S2019472	*Pseudomonas simiae*	Glacier	Arctic Yellow River Station
CCTCC S2019473	*Pseudomonas canadensis*	Glacier	Arctic Yellow River Station
CCTCC S2020027	*Pseudomonas nabeulensis*	Flowers	Arctic Yellow River Station
CCTCC S2021003	*Pseudomonas marginalis*	Glacier	Arctic Yellow River Station
CCTCC S2021058	*Pseudomonas kitaguniensis*	Glacier	Arctic Yellow River Station
CCTCC S2021174	*Pseudomonas antarctica*	Marine sediments	Arctic
CCTCC S2021176	*Pseudomonas spelaei*	Marine sediments	Arctic
CCTCC S2021179	*Pseudomonas gessardii*	Marine sediments	Arctic
CCTCC S2021183	*Pseudomonas fluorescens*	Marine sediments	Arctic
CCTCC S2021184	*Pseudomonas arsenicoxydans*	Marine sediments	Arctic
CCTCC S2021189	*Pseudomonas prosekii*	Marine sediments	Arctic
CCTCC S2021190	*Pseudomonas migulae*	Marine sediments	Arctic
CCTCC S2021192	*Pseudomonas yamanorum*	Marine sediments	Arctic
CCTCC S2021193	*Pseudomonas piscium*	Marine sediments	Arctic
CCTCC S2022057	*Pseudomonas frederiksbergensis*	Soil	Arctic Svalbard
CCTCC S2022058	Pseudomonas migulae	Soil	Arctic Svalbard
CCTCC S2022059	*Pseudomonas mucoides*	Soil	Arctic Svalbard
CCTCC S2022060	*Pseudomonas silesiensis*	Soil	Arctic Svalbard
CCTCC AB2022412	*Pseudomonas* sp. G11	Soil	Arctic Svalbard
CCTCC S2022062	*Pseudomonas yamanorum*	Soil	Arctic Svalbard
CCTCC S2019477	*Pseudomonas proseki*	Glacier	Arctic Yellow River Station

CCTCC S2022056	*Pseudomonas cyclaminis*	Soil	Arctic Svalbard

∗ The species showing the highest 16S rDNA gene sequence similarity

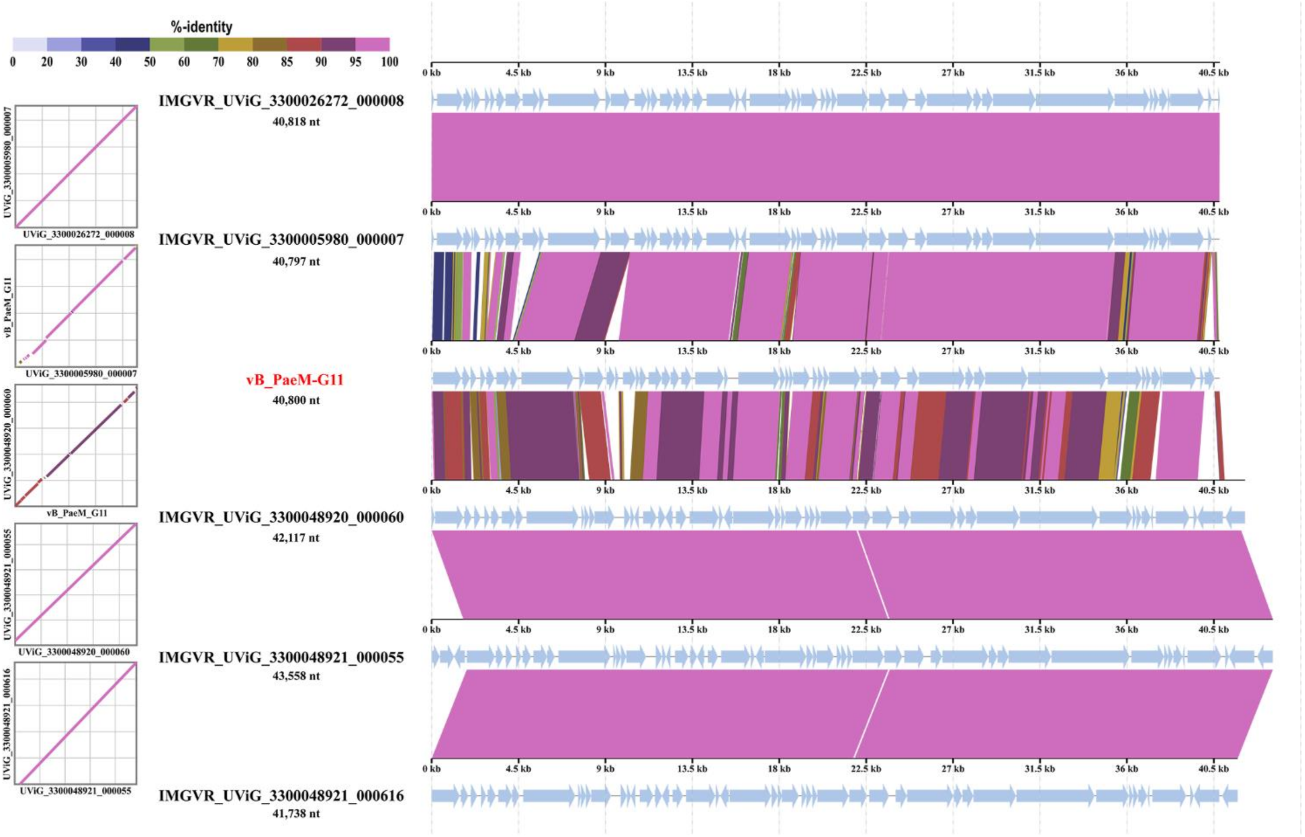

Figure S1. Genome comparison between vB_PaeM-G11 and five UViGs from IMG/VR. In the alignment, high-similarity regions detected by tBLASTx are color-coded based on the reported %-identity. In addition, dot plots summarizing these high-similarity regions are shown beside the alignment.

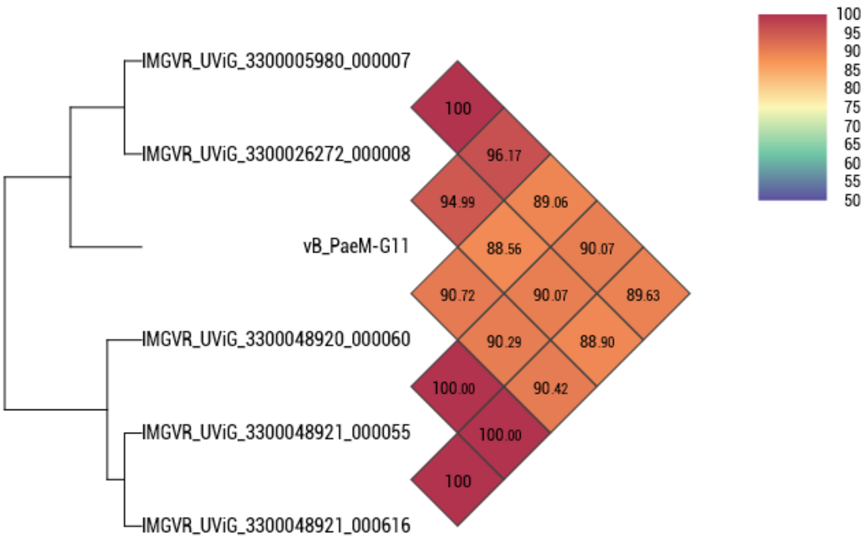

Figure S2. Heat map based on OrthoANI values calculated using OAT software about vB_PaeM-G11 and five UViGs from IMG/VR.

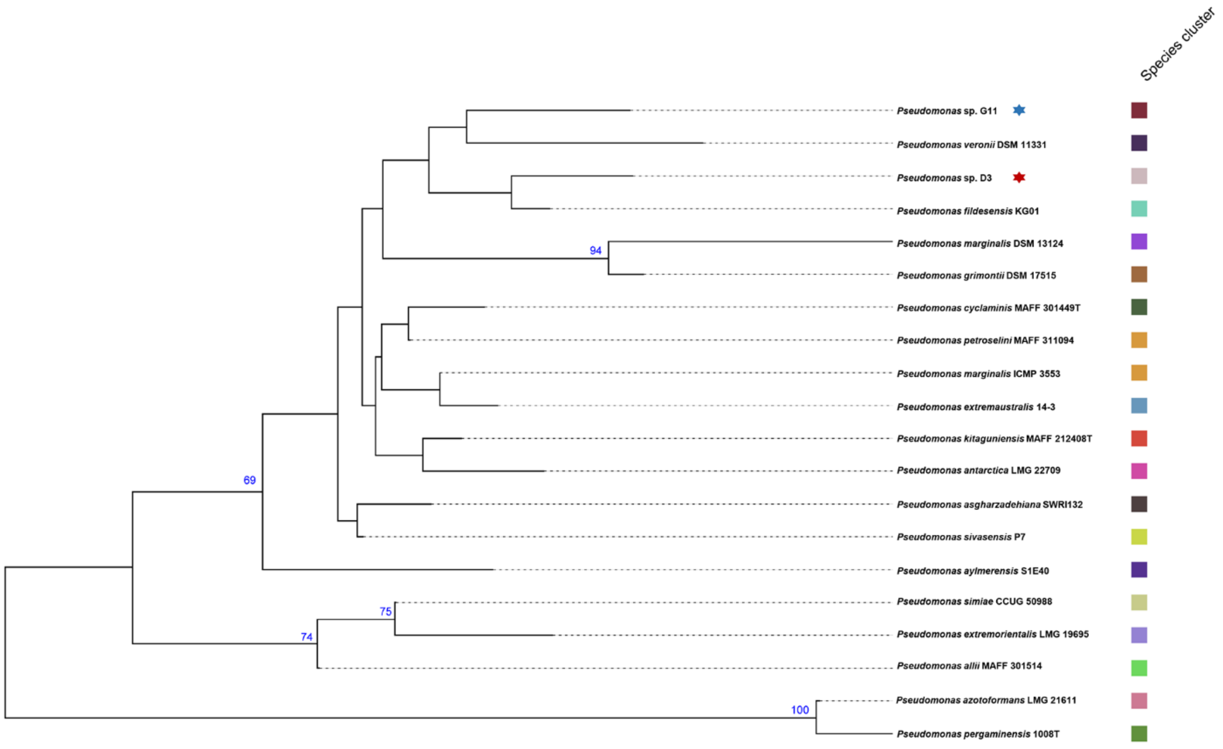

Figure S3. The phylogeny tree of GBDP based on 16S rDNA gene sequence of *Pseudomonas* sp. D3 and *pseudomonas* sp. G11. Tree inferred with FastME 2.1.6.1 from GBDP distances calculated from 16S rDNA gene sequences. The branch lengths are scaled in terms of GBDP distance formula d5. The numbers above branches are GBDP pseudo-bootstrap support values > 60 % from 100 replications. The tree was rooted at the midpoint.

Supplementary Data 1.

Region 6 DNA

CGTATTCTGTCAGAGAATGCTTAAAGTTGACCACCAATGAGCCCTAAGCCACGCAGCGTAAGGCTCTGACCATGAACAACCCCGCGAATCCCTTGA
AGATTTCTAACAGTTTGTTAGAGCTTGCCCGTCCTTATCTGTACAGGAGGGAGGGTCGTTATTCCCTAAGAGTCCGCGTGAAGGGCTCCAAGGACA
CCTGTACAATAGCCCTCAAGACAACGAACAGGCACACAGCATTGGCTACAGCAACACGTCTCCTAAGTACCCTTAAGACATTCCACTTGGACCGCC
TGGACGCTACCAGGGAGGAACTGTGGGAGCGCCTACGGGACATCGCTGAGGGAGTCTTAGAGGCTCGCACTGAGTGGAACCCTCACGGCGGCAT
GGGGCTTACCTACTCGGACATTAACGACAACCTGAAGGACATTAGCCTCACAGAGCCACTCAATGTTGAGCAGGCCAAGATAGTGCCCTTTGCACA
GAGGGTCATGTGGGCCGCTGAGCGCCGCGCCGAGGGTGACTTCGCGCCTATCTTGAAGATACTTGAAGAGATCAAGCGGGAAGCTGGGCATCAGA
CTATTAGCGTCCCTACAGAGCGTCCTGTGGGTGTGCCTGAAGCGGCTCCCGAGGTTGTGCAGGACTCTGGCGCACTGACCTTTACGAAGCTCGCTG
AGCTGCACATGTCTGAGCGCGCTAATGATTGGCAACCGAACACTCAGAAGACCAAGAAGACGGGCTTTAAGACGATTGGCTCACTGCTGGGTGAC
TTGGACCTGCGCAAGCACACTCGTAAGGACATGACGGACCTCAAAGAACGTCTCATGGATGGTCGCAAGGTATCTACAGCCAACAAGCTGCTGAT
TGATCTTTCATCGGTTATGACCTGGGCAGAAGATAACGGCTACATCGATAAGAGCTACGACAAGGGCTTACTGATTCGTAAGGGCGCTGAAAGTGA
GCGTGAGGCATACAGAGCGGATCAAGTGAAGGCCATTATGGAATACGCCAATGGTCTCCCGCTGAACTCCTGGCAGCGCTGGGCGCTCTCTCTTGG
AGTAGTCACAGGGGCTCGTATCGGTGAGCTTCATCAGTTGACCACATCAGATGTCTTCAAAGACGGTGATCAGTTGGTCATTGATGTAAACGAGAA
CGAGGGTAAGACCCTAAAGAACAAATTTAGCGTCCGACAGGTTCCTGTGGTGGATGCGTTGGGCTTCGATGTGGAAGCCTTCGAGAAGTTTGCCC
AGGACGCCCAAGGATCGCTCTTTACGATGTCTCTGAGCAGCTTTTCATCTCTCCTTAACGAGCGCCTGCGGGCCATCCTGATGCTTGAATCAAGTAG
CGGCCAGTCCTTCCATTCGCTGCGCCACCACCTTGCGGGGGCCATGAAGGCCGTAGAGGTCAATGAGGGCACAGCTCAGGGTGTATTGGGGCACA
GCTCAGGAAGTATTACGTTTGACCTCTACGGGTCTGGTAGGAGCGTGCAGGTACAACGTATGGCAGTCGCTATCAAGGTTGCGCTCGAAGAATGCT
AAAAGGCGTTTGATGTATCATGTGAGGCTTTCTAAAGGGAGCTTCATGTGATGCGCTGTGGTTATGTTGGTGTTGCGGGTGCGCTGGGTCTTTTGCT
CCTGGCTGGCTGTGGTAGTCACTCTTCGAATCTCCAAGTCGGCAATGAGCAGTCTTACGCGAGCCTAAAGGACCAGCGTGAGGCTGTACGCACCGT
GAAGGTCTATGAGGCTATGCCTGCTGGTGCTGAACCACTGACCACTGTGTCTGCTGGACGGTGCCATAGGAGCTTCGTAGAGGAATCACCTACAGA
AGCCAGTGTGTTGATTGACTTGAAGGTGGCTGCGTATGCTCAAGGCGCTGATGGAATCACAGGCGTAAAGATCACGAAAGAGTCCGGCCTTAACC
GTAATTGTTGGTACGTCCTGGGAGGAACAGCCCAGGCGTTTACGCTCCGTTCTAAGGATTGATTACAGATGCAATGCCCACTGCCAGAACTCCACAT
GACATCCGAGCGCCTTCACGAACTGGCGCCGGTACGTACTCACTCCGACCTGATGCGTACCATCCTCAAGGGGCTCTCAGAGCTTGCACAAGGTG
GTGTCATTACACGGAAGGGCGGGGCGCTCGGGGTATACTTTGCAGATCAAACGGTGATCGCAGAAGAGTACCACTATGTGGACTCGATGAATCGGG
AGCGGGTCGCCCTGAAGTTCGGGTCCTGGCTGTGCCTGGCACTGGCGCTCAAGGTCTCCCGAGAGGACACGCGTTTGGTTCTCCTTCAGGCCGTT
AAACGGCACCCGATCTACTGATGTGATTTAAAACACCCACTAAAAGCGCACTCAAGGAGCCCGGTCAGTCCTCCCATGGATTCACCGGGTTTTCTT
TTGTGCAGATAAACGCATTTGGTGTGTTGACATGAACACGTTTGGTGCGTAGATTTATCTCACACCAAGGCGGTCCAGCAGGAAGCCTTAAATAGAT
AAACACATTATGTGCGATTAATAGGGTTCAGACCATGCAAGCAATCACCATCAAGTACCTGCCAGCTACCAACACCAAGGGCTCCCGCTTCAAGGC
CACTGCCGCCGCTGGCTCCATCACGGTGGGCTATGACTACGGCCTTAACACTGAAGGAAACGTAAAGGCCGCTGTGAGTGCGCTTCTCGCCAAGC
TGGGTTGGACAGCGGCGAATGGGTACACCGTAGGTTGGGCTGTAGGCCAGATGCCAAGCGGCGCGTGGGCTGCTGTTGCCCTGGACTCCTACTCA
ACGATTTAACTCAGTAAGCCACAGCCACCTTTAAGAATGCCCAGGAGGGCAAAAACCGATGACTATTTACAACCTGACCAACTTCACTTCCGCCGC
AGTCGCTAACAAGATCCTGGGCGCGATGCAAGACGCCATCAAGACAGGCCTCGCGCAGTACGTGACCAACCGCAATGGCCGCTTGTGGCTTCGTG
TGGACATCAAGCGGGACGACCACGGGCGCCTCTACTTCCAGTTCCTCACAGACACCATGCAGGAGGTCGGGCACCACCTCTTAAAGGCCGCTCTG
TTCCACTGGGCCCGTGAGGATGAACGTGAGTTCTCAGGGCTCCACGCCGAGTTGTACCGGCTCCCACAGCATCCTCTGGATACCATTCTGGACGCC
CAGGAAGCGGAGAGGGCGCGCCGAGAATTCTCATGGGGACGTAAGGTCGCTGAGTGGATTGGATACCGCTTGAACTGGCCTGGTATCGCTCTCTG
ACAGAGGTCTTTTACTAACGTATATGCATCAGTGCATAGAATCTGTTGCGAAAATGCATAATACTGTATGCGCAACCAGTGTTTAGGATGGAGAAAC
CGCATGAACACAGCAGGACAGAACCACGTATTGAGTAGGGCTCTCACGGCCTATCCATCTATGGACAAGTACCACACGGACCTTCGGGACGTTCTT
GACACCATCAAGGACATGGCCGATTACGCTGAGGACCAGCACCCGATAAGCCTGGGGGATTACATCGCGGCGTCAGACTGGGCAGGTGCATTAAG
CGCGATCACCCATTACGGCTGCGGGGTCTACTCGTTCCCGTACCTCTCTGAGGCGTTCTGTAAGGACCTAATGCTTGAGCTGGATTGCATGGCGTAC
AGCGTGAACGACGATGAACCAGAAGAGGCGCAGATTCCTGAAGTCACCTTTCTGACGGAGTGCCGCCCTCTCTTTGATTGCTTGCACAGCCTATGG
GCAAACGCGGGCATCCCTTTGGCAAAGGTTCTGATGGGGCAGGACCCGAGCGTCCTCAAGACCATCCAGGCCGCTCAGTACACCGTTGAGAACAC
CCCAAGAGGTCACTGGCATAACGATAAAGATTCAGACGTGACGTTGGTAGTTGCTCTAACAGCAACCCATACAGGCGGCGGGACGTTAGTTCATAC
CGGGCCTTTCAGCGAAAACATAGTGGTCCCTCAGTTGCCTGTAGGACACGCGATGTTCTTCAACGGGAAAGCGCACAGGCACTACGGGATGCCCG

TAACGAGCGGGGAAAGGAATCTGTTAGTGCATTGGAGTGAAGTTAAATGACACCGCCATTTTTTTCGTGCCTGTTAGGAATTGAATGCGTACTTGG
GCAACACCCAACTAAGGGCGGCTCAAAGGTCAACAGTTACAAGGAGAGTAGCACAGTGACATCATTTGGCAACCCTGTAAGTCGTATTCGGTTTC
TCGCACAGGTTCCAAGTTTCCAAGACTTTCGCCGCCGTCAATATGCTTCCAGCACCCAACGCTGTGAGTTCACATTAGGGCTCGGTCCATGCGGGC
GGACTGCAACGCGCCTCGGTTATCAACTGGACAAGGACTTCCTCACGATCCTTCAAGAGTGTGCAGCTCCCGATGAGGGAAAAATCGAAGTCAAG
AAGTTTATCTACTTAGTAAGCGAAATAACTGGTCGGATAGAGGTTGCTTCCGTGTAACGATTCGCCCTCGGGTTTAAACACCCACTAAAAGCAGCCC
TGCTGTTCCCACCCTATGAGCCTTTAACTTAGAGACTTCTTTAAGTCCCCTCTAAGGACCCTCTACGCCCTCAATTCAGTAAATCACCCAACTAACCA
ACTTCCATAACTTTATTGGAGGCCCAAGTGGCCTCTAAGGGATTCTTGTGTCCGCAGAAAGCCAAACCATCCAACTAACCAAGCATGACTTTAGCG
CAATCACTGATGTTTCATCCTGGGCCTATGAGACCCTTTCTAATATCTACGGCCCCGACCTTGCGGCGGCTCAGTTAAGTCTTGAGCATGAAGCCTAC
ACGCTTGGTGAAGATCACTTTAAGAAAGTCCTTGATCGTTCTATTGATCGTAATGAGTTCGCGGACAACGCCACAGCTAAGCCTGTGCTGGCCTCAC
TGATTCCCCTGATGGCTAAGGCTTTCGAAGACTGGGTTGAGCATCAAGTGAATAAGGTGAAGCGAAAGAATATTGGTCTGCCTTATCTGCAATTGGT
CAAGGCTGAGAACGTCGCGGCAATTACCGTCAAGACAGTTCTTAATATGGTCGCCAAAAAGGGGCCGCTGAGTGTGCAGCAGGTTTCAGTATCCCT
CGGTAAGGCGGTTGAGGAAGAGGCCAGATTTGGCCGTATCCGCGAGCAGGAGGCGGAACACTTCAACAAGCGTATCCGCCCAGCACTCAACAAG
CGCAACGGCCACACCTACAAAGTTAAGTTCATGGAGAAGGTCGAGGCTCACATGATGGCCGCTAATGAGCTGACCACTAAGTGGACCTCCTGGGA
CTCACTGGACAACTACGTCACCTTTCATATTGGCGTGAAGCTCCTGGAGCTGCTCATTGAGTCGACTCAGTTGGTCGAGATGAAGCGGGAACACGC
AGGCAACGCAAAGTTAGACGGCGAGTTCATCTACCTAACCCCTGAGTGGGCCGAAAAGCTATGCAACCGTGCATATGCACTGGCCGAGATTACTCC
GCGTTACCAACCAATGATCGTACCGCCACGCGAGTGGACTGGAATGATTGGTGGTGGTTACTGGGCGAAGGGCCGTAAGCCTGTGCCGTTGATTCG
GGTCCGCTCAAAGAAGGCTCTACAGCGCTACAAGGACGTGTCGATGCCCGAGGTCTACAAGGCCGTGAACATCGCCCAGGCGTCCGCGTGGAAG
GTGAACGCAAAGGTCCTCGAAGTCGCCAAGGAGGTGATGCAGTGGAAGCATGTTCCAATCGACAAGTTTCCTACAGCCGACAAGGAAGCCTTACC
GGTCAAGCCTGCGAACATTGACGAAGACCCCGAGGTCCTCAAGGTCTGGAAGAAAGCCGCTTCGACTGTGTACCGCAAGGACCGGGCCCGAGTG
AGCCGCCGTCTGTCCTATGAGTTCATCTTAGAGCAAGCCACAAAGTTCTCTGAATACGAGGCCATTTACTTCCCGTACAACCTCGACTGGCGGGGC
CGTGTTTACGCCATCCCATCGTTCAATCCACAAGGCAACGACCTGACCAAGGGCCTCCTGATGGCTTCCATTGCGGAGCCTGTAGGCAAGGACGGT
ATTGAGTGGCTGAAGATCCACGGGGCCAACTGTGCAGGGGTCGATAAGGTCGACTTTGGGCAGCGCACGAAGTGGGTAGAGGACAACGAAGAGC
TGATCCTTGAGATTGCTCGGGACCCACTGGGCCAGCCTGAGTGGACCAAGATGGATAGCCCTTTCTGCTTCCTGGCGTTCTGCTTCGAGTGGGCCG
GTGTGGTTGAACACGGTGAGCTGTGGGAGTCGGCACTGCCTATCGCATTCGATGGGTCTTGCTCTGGTATTCAGCACTTCTCCGCGATGCTCAGAG
ACGAGCGGGGCGGTCGTGCTGTGAACTTGTTGCCTTCGGATGAGGTGCAGGACATCTACCGCTTGGTTGCTGAGGAAGTCATTGAGGCGGTCAAA
CGGGACATGCTTGAAGGCACGGAAGACAGCACCGAAGTGGTGACGAACAAGAAGACCGGTGAGATTACAGAACGCCGCGTGTTGGGTACTCGCT
CAATGGCCGCTGGTTGGATGGGCTACGGGATCAGCCGGAAGGTGACTAAGCGTTCCGTAATGACTCTGGCGTATGGCTCCAAGGCTTTTGGGTTCA
CCGACCAAGTACGCGAAGACATCATCCAACCAGCTCTTGACGCTGGTGACGGTGAACAGTGGTTCCCTGATGCACAGCAATGTGCCCGGTATATGG
CGAACCTCATTTGGGATTCTGTGAGCTTGGTCGTAGTTAAGGCCGTTGAGGCCATGTTGTGGCTCCAAAAGGCTGCAAAGCTGCTGGCTACTGAAG
TGACCGCCACAAAGAAGACCAAGAAGAACCCCGAGCCCGAAATCCTGAAGCCTTGCATGCCTGTGTACTGGGTGACGCCTGACGGGTTCCCTGTG
TGGCAGGAATACATGGTGCCTGTGCAACGCCGGGTCGACCTGATCTTCCTGGGTGCTGCTCGCTTGCGCTCAACGATCAATGTTCGGGATTCCAAC
GAAATCGACGGGGCCAAACAGGAATCGGGTATCTCTCCGAACTTCGTACACAGTCAGGATGGGTCCCACCTGCGCAAGACAGTCGTTAAGGCTTC
CGAGTGCTACGGCGTGGAGTTCTTCGCGTTGATTCACGACAGCTTCGGGACCATCCCAGCGAAGGCCGGAGCGATGTTCAGGGCTGTGCGCGAAG
CGATGGTTGAGACGTACACAGAGAACGATGTCTTGGAGGACTTCCGTCAGCAGTTCATGGAGCAACTGCACGAGAGCCAGCTTGAAAAGATGCCG
CCGATTCCACAGAAGGGGACCCTTGACATCACCAAGATCCTTGAGAGCCAGTTTGCCTTTGCCTAATTGGCTTACTACTCGGTACGGATTCAAAAA
CACCCACTAAAAGCACAAAGAAACCTCATTGAGATTTAACCGGTCTCTCTGAGGTTTTTCTTTGTCTGTGCGATTTAAAACACCCACTAAAAGCAC
CACAAACCTTCCCAATATTTTAGGAGTTCTTTCCGCTATGCAATCCACTAAGACAATTGAACGCAACACCCGCACTTCCAAGCGCCGCACTGATGAC
GAGATGGAAGCTGTACGGGTCCGTCGCGGCAAGTTGAATAAAGTGCAGCGCCAAGCCCGCCATAACTGGGAGAGCGCAGAATGATCAACGTAAA
GACCTCCTATTCGGCTCTCGATAAGAAGACCTTTTGTTTCGACACAACGTTGACTATTGAGGATGCGTCTAAGACTACCCGGCAATTGGTAGATAAG
CGCGAATTGCGCTCCCTTCGTTTCATTACGGCAACTACGGACGCAAAGCGCTGCTATCAGTACGCGAAGTTTCTCAACGACACCGTAGAGATGCGA
GTGCGCCGAGCGTTCGCAACGACCTTCACGAAAGTCCAAATGCTGACCGGCAAGGAAGCTAATTAATGAGCAAGGTTCAAAGTGTAATCCTCGAA
ACCAGCCCGCACCGACCAGTCAACTTCAATACGAAAGCCGTCACTAAGGTTCTTGATGAGACCTATTTGATTGCTGATGTTAAAGAGGATGGTGTC
CGTTTGAACCTTTGTGTTGAGCCTTTTGGTTCTGTGCATCGAGTCCATTGGTTGAGCCGGGAAGGTAAAGAGTTTCCTGCGTTTAATGATCAGCTTC
CTTTAGCTCTTGACCCTCGTTGGTCAAAGTTCTTCGAGTTAGACGAGGCTTTGTTCTCTGAAGGGTTCATGCTGGACGCTGAGCTGCGTATTGATGA

CCTACCGTGTAAAGACATCGCCGGGACTCTTCGCCGTCACCAGCCTATCGACCTGAGTCGCCTGAAAGTAATCGTGTTCGGCATTGTGCCAATGTCT
GTGGTCCGTTCGGGCCAAGACTATGACGTGACCCACAGTGTCATGAAGTACCACATTGAGTACCAAGTGAAGTTGCTGCAAGAGCGCTTCCCTGA
GATTGCTTGGTCGGTTATTGAGTCCATGGACTGCTTCAGCATGCAAGAGGTTGACGCTTTTTATGAGTCCGTCCGTGAGCGGAAGTTAGAAGGTGT
GGTGCTTAAAGACCCTAACGGTATCTGGAAGCGCTCTAAGCAATCCGGTATGTGGAAGATGGTTCCCGATGACAACGAAGACGGAAAGGTTGTGG
GTCTCGTTTGGGGCACTCCTGGGCTGGCTAATGAAGGAAAGGTGATTGGCTTCGAGGTTCTCCTTGAATCCGGCCATGTTGTGAATGCCTGCAAGA
TCAGCAAGGCACTTATGGAAGAGTTCACGGCCAAGGTTAAGGAACATCAAGAGGAACGCTCGTATACCGACTACCTGAACCCCTACCACGGCCAC
ACAGTCCGCGTCACGTTCATGGAGCGATATCCGGACGGTTCTTTGCGTCACCCATCTTTCGATTCTTTCCGTGGCATCTCTGAGCCAACTATTAAGG
AGTAACCAATGAAGTCAGCTCTTATTGTTTTCGGCGTGGTTTCGGTAATCGGTCTCGCAGCTTGGTACGAAGTAACACTTTGGCAGGAGTGCCGTGT
AGATCACTCGTGGTTCTACTGTCTCCGTGTCCTCTCTCATTAAGGAGTAATGCATGACCCTTCTGCAACTGTTTTTCCCTATCGTCACCGCCGCCCTT
TCCGTGGCCTGCTTCGCCTGCTGGAAAACTAACAAACCACTTCGTGAGAAGGCTAAGGAAGAGGCGCGCATAGTTGCCGAAAAGGCCGCTAAGG
AAGTCGCTGAGGGGCGCGAGAAGGATCGCCGCAACAACTTCCGAAAGGAACTGTATAGCGACCTTGAACAGATCATTGCGACCCCTGACATGTTG
GTAGACCGTCGATACGGCCACCGTGAGCCGCTGGGGCACTATCGCTGGAAGAATCTTGCTGAGTTCATTCTGAGAGTCAATCGCCTCAAGGATCAA
AGCGAAACTATTGCGTACCTTGACCAGAGAATCTTAAAGCAATCTGCTCTGATACGGGACATGCAGTTGAATCAGCGGGACATCATGAAGGCTCTG
CAAATGGGCAACCCAGTGGAGTAACTAATGGAAGAGCACGAAAAGTTTATTCGTGAACATCTTGTGGCTTTACAGCGTGAGTACATGGAGCGGGC
TAAGCCTTGGGTGGACCAGTTGGTTCAGATAGAAAGCATGAAGCCTGTCCGTTACCTGCTCACTCTTGAACAAGCTGCTGCAATGGGGATTTACCC
GAAACTCTAATAATTCTCTAAACCATATTTAATGGAGGACTCCTATGTCTCGCTCCACGCCTGCGTTCGCCTATCGTTTGGCGCAGTCCGCAAATGAT
CGTGCATATAACGCTGCACTATTTACTTCTCAGCGCTCCCAACGCCATACATCGACACCGAAGCCTAAAGAGGCTCTGTGGGTTCGCATAAAGAATC
GTCTGTTGGACATCTGGCAGAAAGTAAAGGAATACGTGAATGACTGACCTCCTGTGTCTTAACAGTATTCGAGGGAAGTCCGGTAAGGATACTTTG
ATTAGTCTGTTGGAATCTGAGGGCCGCAAAGTTTTTCGTGTGGCTTTTGCGGATGTCCTTAAACGAATGTGCGCCGAAGAACTGGCCGGTCGCCGT
AAAGGGCTGGCGGTCATGTTTGAACGTGACATGCACAGTTCTGCTAAGGACAAGCCGTGCCCAGGTATGGCCATCGAAGAGCTGGAGCGTTCCGC
TTATACGGATTTCCTGAAGTCCCGTATGGAGAGTCGTGAAGACTTTCAAAAGCCTCGCTCTTTACGCTGGCACTTGCAGACCTTCGGGACTCAATAC
CACCGTGAGTTCCTCGGTAAGCCTAACGTCTGGTTTGACTTGGGCATGATCGAAGTTCGCAAGGCACTCGAAGTCGGTACTTACGACCATGTTGTG
GTTACTGACATGCGAATGATCAACGAATACGAAGGGCTGCTTTATGCGCCTTCCGTTAAGACAATCCGAATAGTCCGCGATTGGTTCATTCCAGGCG
TAGACGATCAGCCTTATCACATCTCCGACATTGCACTAATGGCCCATCCCTTCGACGCCCTTGTGGTGAATCGACTGGGCGAGCCGTGGGCGATGTT
GGATCAACTCAAAGAACAAGGAGTAATCAAATAGTGGAACTTGCGCATAAGACCTTTAAGGCAACAGTAGAAATTGACGATCACGTAGACGAAGT
AACTATCTACGCGGTCAGTATGGACGCTGCATGGGCATCTGCTGAGGCCCGTTTTAGCAGTGGTGCCCGTGTAACTCGTATCCGTCCTATGGTCGCT
CCGAATGTGGATCGCTTTGGGGTGACTCTGTGAACCTCTCACAGGGCAACGGTCGGGCAAAGCCTGACGGCTTCCTGCACCTGAACAACTTCAGC
CATGTCCGGCAGTCGGGCCTTGCTGGTGTTCTCTATGAACGCCTAATGACCGTAAAGCAACAGGAGCTGGTCGAGCTGACTTTGTTGGAGCTTGCG
GGTGCCGAAAGTCATGCACACTTCAGACATGACGTATGGAGATTCAAAAAGTCGTTCTTGAAGGAACACTTTGTCCATGTGGTCTATTCGGTATATC
GCTCGGTGCGTAAGTCGCCAGCACAAGAAATCTCCATGGCTATTAGTCGTGAAGAACTGCAAAGCGAATCCCGCAAGGTTATTCAACCGAGCTTCT
CGTAAGTTTTAAACACCCACTTAAAGCACACGGACTATTCACTTGAGACTTAAATGTCTTGAGCGGACTCCTGCGCCTTCAATTCAGACACAACAA
GGAGACCCATAACTATGGCAGGTCCACGTAAAGAAATTCTCACCACTCCTAAAGGGATCGCGGAACCGTACTGCGCCCTTCAAAAGCCGGACTAC
GGCAATCCAGAAAAGGGCTTCGGCAATCCCCGTGGTCTCTGGAAGGTTGACCACACCGTTCCTTCCCGTGAGGCTCAGCCCCTGATCGACAAGAT
CGTTCAGGCGCACGAAGCCAACTATAAGAAGCTCTTGGCCGAACACGAAAAGAACCCACCAGCGGTTCCTCGCGGTAAGAAGCCTCTGCTGCCCT
ACGAGGGCGATATGCCTTTCGTGGATAACGGCGACGGCACTGTGACCTTCAAGATCAAGGGTTACGCCTCGTACATCGACAAGAACACCCAGGAG
AACAAAGCGATTCCCCTGAAGGTGGTCGATTCCAAAGGTAAGCGCATCGACAACGTACCGGCCATTGCTGGCGGCTCTGAGTTGAAAGTGAAGTT
CACTCTGTTCCCGTATGGCTGGACCGCTGTGGCCGGTGCTTCCGTGAAGCTGCAAATCGACAGTGTAATGCTGCTGAACCTGAAAGAGTTCGAGGG
CGGCAACGACTGGGCTGATGATGTGGAAGAGGGCGGCTACTCCGCTGATGATTCCGGCGAATGGGAAGAGGGCGAAGAGTCCGGCTCTCGCGGT
CACGCTGACGAGGAAATCCCAGACAACGACGGCGACTTCTAACCGTGGCCGCATTAGGCCGCTACGCCGGACCCCGAAGGGCTGCAAGTTCCTTT
CGGTCAGGTCTGGAAGAGAAGAATAGCGAACTGATGGACAAGCACGGTATCCCTTACACCTTTGAGCAGCATTGGATTAACTACACGATCCCTGCA
CGAGTCGCTAAGTATCTCCCGGACTTCATCCTGGGGAACGGCATCATCATTGAGTGTAAGGGCATATGGGAAGTTGAAGACCGCAAAAAGCATCTG
CTACTGCGAGAACAATATCCTCAGCTTGATATTCGATTGGTCTTCTCATCGAGCAAGGCGAAGCTCTATAAGGGCTCACCCACAACTTACGGGGCGT
GGTGTGAGAAACACAATATTCAATTCGCAGACAAGCTGGTCCCACTGGCTTGGATGAAGGAGAAGAAAAAGCAGATTCCTGACGGAATTCTGAAA
GAGAAAGGAGCGTAACTAATGGCTCGTGTCCAATTCAAACAGCGGGAGGTGACAAACCTTATCGTTCTTCATTGCGCGGCTACACGTCCTTCTATG

GACATTGGCTTGCGTGAGATTCGTCAGTGGCATGTACAGCAAGGCTGGCTTGACGTTGGCTATCACTACATCATCCGTAGAGATGGCACCGTGGAA
ATCGGCCGTCCTCACGATGTCGTAGGGTCCCACGTTAAAGGGCACAACTCAGAGTCTGTTGGAGTGTGTCTTGTAGGAGGCGTTGACGACAAGTT
GAAGCCTCAGAACAACTTCACGGATGCTCAATGGAAAGCCCTGGACCTTTTGGTATGGGAAACCCTTGTGCCGCTGTATCCGAACGCCACTCTTAA
AGGCCATAGAGACCTCGACTCTGGCAAAGCCTGCCCGTCCTTTGACGTGGCTGAATGGGCATTTAAGAGAACCCCTGCGTAATACCCATCAAGCAA
CTGTAAGGCGGGCCTAATGGGTTCGCTTTAACAGCAATTTCACCTCTTAACTTAGGAGGATTACTTATGTCTGTTAATCGGGCAACTATGCAAGGGG
CTTTTGACCTATGTGAATTGTTCCAGAAAGAAGGCGTCCCCAGCATTATCGCAGGTGGTGCAGCCCGTGATATTTACTTTGGGGTCACTCCTAAAGA
TGTCGACGTGATTATCTGCTGCGATGACAAACTGTATGCCTCATCGCTTCTTGAAAAGGCAGGGGTCGCGCACTCTGTCATTCACTTCTACAACGAG
TCTTCTTCCGATCGAATCATCGGTGTCTTCAAGATCGAAGGCACGAACATCGATGTTTGCCTTTACGACTGCACTGATGTCGGCGAAGCGGTTGACG
CTTTCGACTTCAACCTGAACCAGTTCGTTATCTCAGGGATTCATCACGGGATCGACGGGGCACATGTTCGTTTTGTCGGGCAGACCCACTGGTCCA
CTTTGGCGGCTGTCCGTAAAGACTACTCGCTGAAGCGCCTGGAGAAGATGCAGGCCAAGTTTGTGGACCTCCGCAATCGTAGGGCAACCGGGGAA
GCCCTGGAGGTTCCTGTAGGCGGTGTTAATGGACCGTTCTGAGGACCTACCGGAAAGCGAACTGCTCTACAAAGGTCCTTGCACGAACGTTGAAG
AGTGTTCATCCAGCGATGGCATGGCGACTTACTCAGACGGCCATACCTTTTGCTTCGTGTGCAATCACCACACCCCAGGCGATGGCTCAGAGGGTC
ACAGGACCCGAGCGAACACCTTGCGTGCCAACGGCACAATGGCGATGGGAGAACACCAAGGCCGCTTTCAGGATTTGCCTAAGCGTGGCCTGCA
ACAGGCCATCTGCAAACAGTACGGATACTGGGTAGGCAAGACCCACAGTGGCAAGAACATTCAAGTCGCTGACTACCGTGACGAACATGGGAACT
TGGTAGGTCAGAAGGTCCGCGATGCAGACAAGAACTTCTCATCTACGGGCAAGCATGGAGCTGACTGCCTCTTCGGCAAGCATCTGTGGTCTGGA
GGCAAGAAGATCATCATCACGGAAGGTGAGATTGACTGTCTGACCGTGGCCCAATTGCAGGGCGGGAAGTATCCCGTGGTGTCCCTTCCTACAGGC
GCTCCGAGTGCCCGTAAGGCCTGCGCTAAGAACTACGAGTATCTCGATACGTTCGACGAAATCATCCTCATGTTTGACATGGATGACGTTGGCCGTG
CAGCGGCAATGGATGCTGCTGAGGTTCTGCCAGCGGGCAAGGTGAAGATCGCCGTACTGCCCATGAAAGACCCGAACGAATGCGTTATGAACGGC
CAAGCGAAAGCCATCATGGATGCAATGTGGAACGCCGCGCCTTTCGTTCCCGATGGCGTGGTCTCCGCGAAGTCCCTGAAGTCCCGCATTAAGAAC
AAGCAAGACATCCCGAGGATTCCCTTGGCGGGTCCTGCTGAACTGCGCCGTATGACCAAGGATGCACGGGCCGGTGAGCTGCTTATGGTGACTTCC
GGGTCAGGCATGGGTAAGTCCACCTTTGTGCGCCAGAACGTCTATAGCTGGTTCCAGCAACACGGCCTCGAAGTAGGCGTTGCGATGCTTGAAGA
GTCCGTTGAGGAAACCGTTGAGGACCTCGTGGGCTTGCACATGCGCCGTCGCTATCGACAGAACCCTGACGGCACCACAGAGGAAGAGTTTGAC
GCTGCCTTTGACGCCATCTTTGAGACGGACAAGCTGTTTCTCTATGACTCCTTTGCGGAATCCGTAGAAGACCGCTTGATGTCCAAGCTGCACTTTA
TGGTGAAGGGGCAGGGCTGCAAGGTGATCGTGCTGGACCACATCTCTATCGTGGTTTCGGGCATGGATGACAACGGCGACGAGCGGAAGACCATT
GACCGCTTGATGACCAAGCTGAAGACCTTCGCCAAAACCAACGACATCCTCATGGTCGTTATCTGCCACCTGAAGAACCCCGAGAAAGGGAAGGC
CCACGAAGAAGGCCGTCAGGTTTCCATTACGGACCTTCGCGGTTCCGGCTCTCTACGTCAGCTCAGCGACACCATCATTGCCATGGAGCGTAACCA
GCAAGGGGACAACCCGAACGTGGTCACGATCAGGGTCCTGAAGTGCCGCTTTACGGGTGACACAGGTGTGGCCGGATATCTGGTTTACAACAAGG
AAACCGGGTGGTTGGACGAGATGCCCGATGGCTGGAAACCGGACAGTGATGACGACTGGAAGGACGACATACCGAAAGAACCAAACCCCGACTT
TTAACCCACTCAAGGAGAAACCCTATGACCATTATCAAACTGTACAAATTCGCTCTGAAGAAACTGGCCGCTGCAATGACCAAGGCCGCAAACGCC
AAGAAGGCTCAAGCCGACTTCCTCGTTAAGGCCAGCGCAAAGGCGGCTGCTGAAGCCCTCGAAGCTGCCAAGGAGAGTAAGCGCCTGACTGAAG
AAGCCGCCAAGGTTTCCGCTCTGATCTAAGAGTGACTCAAGGGTCTCCGCTTTGGGGACCCTTCGTTCAACCTTAATTCGAACACCCAACCAGTCA
CCAATTCCTTTGGAGGATTCACTGTGGACAATCAAAAAGTACGCGCCCACCTGCGCAAAGGTGAAATTGCTGTAAAGCTGCTGGAATCGCTGGGTT
ACTCCTACGACGAATCGAAAGTCCACCTTCAGCGAGAGCCTCGTTGGGTCGCCCCGGCGAAGAGTGAGCTGGAGTTGTTCAAAGAGCAGCTTCAC
GAGCTTCTTACGCCTCCTGAGAAGCGCACCAGTCCTGTACGTGATGGGGCTTCGTTCACTGTGACTTCCCTGCCTCCTGGGCACTACCTGAACGCA
TACATGTATCGCCGGTATGTGTTCTCCGTAGAGTCCTCGGAATGGATCGCGCCGGGTTCTGAAAAATCCGAAAAGTTGAAGGGGTTTTCCGGCTGG
GCAGTTCACTTTGAGATGCGCGGTACGGCCCGTAAGCAAGGCCTGTGGTTGCCGCTGAGCTGCATCAAGGTAACGCCTAATGCTGACTTCTGACAT
TGAGACAGACGGGCTCCTTGAGAACGTCAGCAAGTTTCACTGTGGGGTCAGCAAGGATTATTTCACTGGTGAGGTCTTCGAGTTCGGCCCTGACC
AGCTCAAGGAATACATCGCCCAGCTCGAAGCGGAAGCGGCCAAACCGGATGGCCTTGTGGTCTTCCATAACGGGATCAAATACGACATCCCGGTCC
TCGACAAGCTGAAGCGTCAGTACTTCGGTAAACGCCTCAACATCCCCCGCAAGAAGGTCATGGACACCCTTGTCCTGTCCCGACTGATCCACGCG
AATCTTAGAGACACAGACGGTGGCCTTCTGCGTACTGGGCGACTCCCAGGCAATCGCTTTGGGTCGCATGCGTTGGAAGCCTGGGGTTATCGCTTA
GGCGAGATGAAAGGGGAATACACGACCGACTTTAAGGCCCAGTGCCTTGCAGATGGCACCGTTTACACGCCGGGTCTCGAATGGAAGAACTGGTC
AAAGGCCATGCAGGACTACTGCGTTCAGGACGTTGTAGTCACGACCAAGCTCGTTACCAAGTTGATGTCAGACCCGTACTACTTCCCCAGTGGGCT
TGCTCCTAATGGCGAACACTGGATGCAGTCAATCCGTGCGGTTCACCTTGAGCATGACACTGCGTGGACTCTGGCTCAGATGGAGCGCAACGGTTT
CCCGTTCGACTCTGACGGGGCTGAAAGGCTCTACGCTGATCTTGCAGGCCAGCGCTCTGACCTCTTGGTGAAGCTCATTAAGACGTTTGGCTCTTG

GTATCAACCCAAGGGCGGCAATGAGCTGTTCGTAAACCCCAAGACAGGTAAGGCCATTGAGAAGTGGTCTGACGGTCGCCCAATGCCTCGGGTCA
AGTACCCAAAGGTCGGCGGTGTTTACCTCAAGGACGGCAAGAAGAAGGACACACGGGAGACCTTTGCGGGTGCCCCATACACGCCTGTCGAGTT
CGTAACGTTCAATCCGGCATCCGGCGCGCACCTAATCAAGGTCCTCAAAGACGCCGGATGGGAACCGACTGAGTTCACAGACAAGGGCGCCCCAA
AGGTTGACGACGAGACCCTTGAAGGCGTGACCGTTGCTGACTCGGACGCCCAAGCCTGCATCGCCCTGGTGCGTGACTACCTGATGATCCAAAAG
AGAATCGGGATGTTGGCCGAAGGTGATAACGCATGGATGCGACTGGTCCATTCGGACGGCTCAATGCACGGCTCTATCAACCCCAACGGGGCTGTA
ACAGGTCGAGCAACTCACAGCTACCCGAACATGGGCCAGGTCCCCAGCGCCAAGAGTCCTTATGGGCCGGAATGTCGTGCCTTGTTTGGTGCGGT
CTTCGCTCAGAAACGCTTTAAGGGCTGGGAGAACTGCGTGCAGGTTGGCGTGGATGCTTCCGGTCTTGAGCTGCGTTGCCTGGGGCACTTCGGCG
CCAAGTTTGACGATGGTGCGTATGCCGATCAGGTGCTTAACGGTGACGTGCATTGGGCTAACGGTGTGGCTGCTGGCATCTGTCAGGCGACCATCC
GCGACAAGTCCAATCACGACCACGACCGTTGGCGGGACATGGCGAAGACCTTTATCTATGCGTTTCTGTATGGAGCGGGTGACGCCAAGATCGGAT
CAATCGTTGGGGGCGGTGCGAAACGCGGTAAAGAGCTGAAGAAAGCCTTCTTGGAAGGTACGCCCGTTATCAAGTCTCTCCGCGAAGCCCTTGAA
GAATCCCTAATCTCAGACCAGAAGTGGAACCAAGCCCTTCGCAAGTTTGACATCAAGTGGAAGCGCCGTTGGATTAAGGGTCTGGATGGCCGAAA
GATTCACGTCCGCTCACCTCACAGTGCCCTAAACACGCTCCTTCAGTCGGCCGGCGCGCTCATCTGCAAGGCGTGGGTCGTGGAAACCGAAAGGC
TCCTTATGGAAGAACACGGCCTTGTCCACGGATGGGACGGCGACTTCTGCTTCATGGCATGGGTCCACGACGAATTGCAGATTGCTGCACGTACCC
AAGAGATTGGGGAAGTCTGTGTGGCCGCTGCTCAGCAAGCTATTCGCAATGTAGGCGAGTCCTTCGGGTTCCGCTGTGTCCTCGATACAGAAGGAA
AGATCGGGCCGACTTGGCGCGAATGTCACTAACCAATAATCAAGGAGGTCTCTATGGCCGTAACTCTGAATCTTCAACACACCTTTTCGACCCGTGT
TGTTTTCCGCACTGAAGAAATTGCATCGCTTCGGGCCAACTTGGAACGGTACAACAAGCATCGTCAAGAGTTCGCTAAGCAGCTTCTGGCAATGTC
CGACGAGGATATGGTGTTCACTCTTTTTAGTGATGCGTTTAAGGATATTCGTCAAGGCATTCTGAGCGAGTTGAAGGATGCGGGTGTAACTCGCATG
AGCCCCGTTCAGACCAAGAAGGTTAAGTAATGGGTGAATACATTCGGATGCTCTACGCAATCAAAAAGGAAGCCCGCACGTTCCAATCTGACTTCT
ACCGAAAGAATGCCCACGGCGTCTCTGAGGCAGCATCGCGGGGCCACATCTCGTGTGTGTCCACTGACGGACGAAACATGGGCGTCTGGTCGCTG
ACGACAGCGGGTCAACTGTTCCTCAAGAAACACGGAGGTGCTGTATGACCCTACCTAATGTTGGTTTTCCTCCAATGTACAAGGAGCCAGTTCGTA
CAGCTTCAGAGGTCGAAGCACTTATAGCTCAGTTGAAGCTGGATAACGAAAGGCTGCGCCGAGAAAACACGCGGTTACGTGAACAAGTCTCTAAC
GCTGGATGGGAGGCATCCGCACTTCACGCTGCTGCTACAGGTGGCTGGCAATGACCGCTCGTATCGGGGTGGCTCTCGACATGGACTTCCTGATCT
TCGAGGCAATGACCGTAAGCGAGCGCGAGCAGGATTGGGGTGATGACATTTGGTCCCTTGAGTGCGACCACGCGAAAGCTCGGGGCATCCTGGGC
GGGACCATTAAGTCCATCTTGGTGGACATCGAAGCGGACATCCGCAAGAAGAACAAGAAGCCCTTTGAGCTGGTGCCGCTGTGCATCATCTCGGG
CTCTGACAACTTCCGTAAGGAGGTCTTGCCGACCTACAAAGCGAACCGTAAAGGCAAGCGTAAGCCTGTGGGCTACCCGGCGTTTGTCGAGGCCT
GCATGCAGCACTACGGTGACAACGCGTTCCGCTGGGATGGCGTTGAGGGTGATGACGTTTGCGGGATATTGATGACTAACCCGGCCCTGGCTGGCT
GTGTCGCTGTGGTCTCCGCAAGTTGCGACAAGGACTTCAACACGGTCCCCGGTATGTTCATGTGGCTTACTAAGGGCAAGATGATGCGCAACAGTG
AAGCCCAGGCGGATCGTTATCACATGTACCAGACCCTCATAGGCGACGTGACAGATGGCTACTCTGGCATTCCCGGTGTAGGTCCTGTGGTCGCTG
AAGAGTTCTTGGATTCCCCGCTGTTCTTCTACGAGGCCTCAAAGGTCATGAAGTCCGGCCCACGGAAGGGTGAAGAGGTTAACTACTGGGCTTCCC
GTGCCCCCACCGAGACGGAAACGCTGTGGGACTGCATGGTCTCGTTAGCTGCATCAAAGGACATGACTGAGGCTGAGCTTTTGCAACAGGCCCAG
GTCGCTCGAATCCTGCGCTGGTCTGACTGGGACCACGAGAACAAGAAGCCGATCTTATGGAAACCGTAGAGGTGTTCTTTACGGGCCTCACTTTCG
GCGTGTTGCTCGGCGTGTTTCTGATTCGACTTTAAAGCACCCACTATAACCATAGGAGACTGAAAGTTTCTGTCTTCTTAAAGGCATCTTTTCGTCTC
CGATAAGGAGGTGTAACTCTTGTGTTAAATGATATTCAAGCGGTAATCAGTGATCCTGAAAACATCCCCGATATTCCGAATGCTTCCGCGCAATACCT
TAACGTCCGCTGCAATGCTTCTTATCTAATCCGTACTGGTGTACTTGAGGACCTCCGTAAGGCGGGTTACTCCGAGTCATTCATTGGCGGGTTCCTTG
AGGGCATGACTGCTGTCACTGAGATTATCGAATTGATGCAGGAGCAGCGGAACACTCCAACCGAAGAAGACTGACAGGAGGTTCTATGTGCTTCT
CAGCAAAAGCCAAACAGCCAAAGGTCGACCCGTCAACTATCGCTGCCCCAGCCCCCGTAATGGAAGAGCCACCTAAAGGTGTCGAGTTTGGTGAT
GGGAGCGATGGTGATAAGTCGGAAACAACCGATGGCATTAAAGACTTGAAGGTCAAAAAGGAAGAGCAGGGTGATGGTACTCCAGCCGCTGCTG
TCGCAAAGGACACCGGACTTGCCAAGAAGGCATCTTCAAGTTCATCCGTTAAACGAGCCCTTAAACGAGGTTAATAACTACAAGGAGGAACTAAT
GGGTTTTTTCAAGAAGGCTTTCAAGAAGGTTTCAAAGTTAACAGGAGGCTTGGTTGGTGGCGGTAGTGATAAGCCTAAAGACCCAGCTCCCGTAG
CCGCTGCTGTTGAGGCGCCAGCTCCCGTAGCCGCTGCTCAAGTCGAAGCTCCTAAAGAGGACACGCAGACTGGCGAAGATGGCGATAGCGAAGC
AGCAAAACGAGCTGCCAAGGCGAAGGGTAAACGATCCCTTTCCGTATCACGCAGCCAGGGGACCGGTCTGAACATCTAAGGAGGTCTATATGGCC
TCTACGGCTCGCACAGGGCTTGGAGAAGAATCGGCGAAGGCAGTCTATGACCGCTTGAAGAATGACCGTGTTCCCTACGAGACCCGCGCTGAAAA
CTGCGCGAAAGTAACAATCCCCTCGCTGTTCCCAAAGACCTCGGATAACGCCTCGACTGACTACACCACACCGTGGCAGGCAGTGGGTGCCCGTG
GTCTTAACAACTTGTCCGCCAAGGTGATGCTCGCTCTGTTCCCGCTGCAAAGCTGGATGAAGCTGAAGGTGTCCGAGTGGCAGGCAAAGCAACTT

GTCACTGACCCTGAGCAACTGGCAATCGTGGATCAAGGCTTGGGTATGGTTGAGCGGATCATGATGTCTTTCATGGAAGCCAACAGCTACCGCGTG
ACGCTCTTTGAGCTGATCAGACAGTTAGCTCTTGCTGGCAATGGATGCCTTCACCTACCGCAACCGGATGCAAGCTCTACGGGCTACAACCCGATG
AAGCTCTATCCCTTGAGTTCCTATGTGGTTCAGCGGGACTCCTTCGGGAACGTCTTGCAGATTGTCACGTTGGACAAGGTGGCCTACGCGGCCCTT
CCTGAAGATGTCCGTAACTCCCTCTCGGATTCTGGCGACAAAAAGCCCGATGAAGAAGTGGAGGTCTACACACACGTTTACCTTGACGATGAGTCA
GGCGACTTCTTGAGCTATCAGGAAGTGGACGGTGAGGAAGTGGACGGCACTGATGGGCAATACCCACCCGATGCGGTTCCATGGATCGCTGTGAG
ATGGACCAAGCGGGACGGTGAAAACTATGGCCGTTCCCACTGTGAGGAATACCTGGGCGACTTAACGTCCCTGGAGAACCTTCACGAATCCATGAT
CAAGTTTTCCATGATCGCCTCGAAGATCGTTGGCTTGGTGAACCCTAACGGGATGACCCAAGTACGGCGTCTTACCAAGGCCCAAACGGGCGACTT
CGTAGCAGGCCGGAAGCAGGACATCGAGTTTTTGCAGCTTGAAAAGACTGCTGACTTCTCAGTCGCTAAGTCCGTGGCAGATGCCATTGAGTCCC
GTCTTGCCTATGTCTTCATGCTGAACTCAGCCGTGCAGCGTTCGGGTGAGCGTGTGACTGCTGAAGAGATTCGGTATGTGGCAAGCGAGCTTGAGG
ACACTCTTGGCGGGGTCTATTCGATCCTCTCCCAGGAGCTTCAGTTGCCGCTTGTGCGGATTTTGCTGAACCAGCTTCAAGCCACCTCTCAGATTCC
CGACATGCCCAAGGAGGCCGTAGAGCCAACTGTAAGCACCGGCCTGGAAGCACTTGGCCGTGGGCAGGACCTCGACAAGTTGACTCAGTTCTTC
AATGGTCTGCAAGCGATTGCGCCGCTGATGCAAGACCCTGACATAAACATGAGCAACCTAAAGATTCGCCTCGCCAACGCCATTGGCATGGACACC
GCTGGGCTCCTGCTCACGGACGCCGACAAGGCCAAACGCCAAGCTGAACAGATGGTCGCCCAAGGCGGTCAGGCTGCTGCTCAAGGTCTCGGCG
CGGGTGTGGCTGCGCAAGCAACCGCAAGCCCGGAAAACATGCAGTCAGCCATGAACACCGCTGGCATTCAAGCGGGCCCTATCGGGGCCTGACA
GAACACCTCAACAATACATTTAAGGAGACCCTATGCGATTCAACATGCTGGCTCTGGCCGTGGGCCACGTTTACCAATCCGCTGACGTCTATGCGTC
CTTCGGCGTGAATAGCGCTGTGATGTCTTCCAGCGATCCTGCTGAACACGCTCAGAACATGTTGGCCCTGGACGTGGCTACCCGTGACGGCGACAC
CTCGATTGACCTTGCGGATGACTCTGAAGAGCAAACCGAAGAGTCCACTGAGGAAGAGGAAGCCAGCGAAGACGAAGAGGGCACCGAAAGTGA
AGATGAGTCGGACGGCGCTGAGCCCGAACAGGGGGAGGATGGCTTTACGCCCCTTGGCGATCCTGATGAAGAACTGACCGAAGCCTCTCAGCAG
CTTGATGAGTACTCCACGGGCTTCGATGAGATGCGCGCCCAGGCAATCAAAGCTGGCCTACCTGCTGACGTAGCGGCCCGCATCGAAGACGAATAT
GAAGCTGACGGGAAACTCTCGGACGACTCGTATGCGCAGTTGGCTAAAGCTGGCTATTCGGCTGGTTTCGTCAACTCGTTCATGAAGGGTCAGGA
AGCTGTAGCAGAAGCCTACGTCTCCAAGATCGTGGCGTATGCAGGTGGCAAGGAACAGTTCGACCGCGTTGTGGCCCACCTGAAAGCCAACTCCC
CGAAGTCCATGGACTCGCTGTATGACGCCATTGAGCGCCGGGACCTGAACACCGTTCAAACGGTAATCAACCTGGGCATGGCAAGTCAGGCAAAG
AAGTTCGGTAAGCAGCCTGAGCGAACTTTGAACCGCCGTAATGCAACACCTGCGGGCCGTGCCCCTGCTCAACAAGCTGAGGGCTTTGCTACTCA
AACCGATATGGTTAAAGCGATGAACGACCCGCGCTATGGTCGAGACGCTAAGTACACCCAGGAAGTTCGAAATAAGGTACTTGCCGCCTCTTGGTA
AGACCTCCGATTTAAAACCCCCACTATAACAGAAAGATACTTTCGGACATCCGTCTGAAGGCCTTTCTGCGCCCGGAGATAACTGAATAAGGAGAG
CTAATACATGGCAAATGCTAAAGGTCAACAGATTGGTAAGAACCAAGGTAAAGGCGTTACCGATGCGGACAAACTGGCTAACTTTCTAAAAGTTTT
CGGTGGTGAAGTTCTGACTGCTTTCATGCGTCGGGCTGTAACCATGGACAAACATATGGTCCGTACCATTCAAAACGGTAAGTCCGCTTCGTTCCCG
GTAATGGGCCGTACTGCCGGTTACTACCTTGCAGCCGGTGAGAACCTGGATGACAAGCGCGGTGACATCAAACACGCCGAGAAAGTTATTACTATC
GACGGTCTGTTGGCTTCGGATGTTCTGATTTTCGATATTGAAAACGCCATGAACCACTATGACGTTGGTGCTGAGTATTCGGCTCAACTGGGTGAGG
CTCTGGCTATCGCTGCGGACGGTGCTGTATTGGCTGAGATGGCTAAGCTGTGCAACTTGCCAGCCGCTGCTAACGAGAACATTGAAGGCCTGGGCA
CTGCGACCGTTCTGAAGATCGGTTCTAAGGCCACTCTGGAAGCTGACGCCGAGGCACTGGGTAAAGCGATCCTGAAGGCGCTCACCGTTGCCCGT
GCGAAACTGACCCGCAACTATGTGCCTGCTCAAGATCGTAAGGTCTACACCAAGCCTGAATACTACAGCGCTATCTTGGCCGCATTGCTGCCTAACG
CTGCCAACTTCTCGGCGCTTACCGATCCTGAAACCGGCAACATCAAGAACGTGATGGGCTTCGAGATCATCGAAGTACCACACCTGACTGAGGGC
GGTGCAGGTGATGACCGTCAAGGCACCAACCGTAAGCACGCGTTCCCTGAGACAACTGGCGGTGACGTACAGGTAGCTCTGGATAACGTGGTCGC
TCTGTTCAACCACCGTTCGGCTGTGGGCACCGTGAAGCTGAAAGATATGGCTCTGGAGCGTGCCCGCCGTCCTGAATTCCAGGCTGATCAGATCAT
TGGTCGCTACGCGATGGGGCACGGTGGCCTGCGTCCTGAAGCTGCTGGCGCACTGGTTTTCACAGCGGCCTGACCTCAGTAACTGGCGTAACGCT
TAGCCAAGCGACTATGACTCTCCAGATCGGAAACTCTAAGTCTCTGACGGCTACCGCTGCGCCTGCTGAGGCCACTAATACTTCTGTCACATGGAA
GTCCAGCAATGAAGCTGTGGCTACTGTGAGTTCTGCGGGAGTCGTTACGGCCGTTGCTCAAGGAACCGCTGGGATTACATGCACAACTGTAGATGG
CTCTTTCTCTAAGAGCTGCGCAGTGACTGTGCCTGCTGCTTCCTAAAACCACCTAAAGCCCCTCATCCCGTTTTCTTAAAGGGTTAGAGGGGCTTTT
TGTTTCTTGCACATACCTTCAGGAGGGCCTAATGGCAAGCCTTGAATCATTCATTGAGTCCGACGATGAGCTGTCAGCAGTTAATGACATGCTTGCA
GCTATTGGCGAGTCGCCTGTTAGTTCCCTGGAAGGGGACCCTAACGCTGATGTGGCTAACTGCCGTCGCTTGCTCAATCAGGTAAATCGTGAAGTC
CAGTCTAAAGGCTGGGCTTTCAATATCGAAGAGGATGCAACGTTAACCCCTGATACTTTCTCGGGGCTCATTAACTATCTCGGCGATTATCTAAAGAT
CACCACCGGAAACTATTCAAATCGCAGCGGGTTTCTCTACGACCGCACAGCGAAGACCGATGTGTTTACGGCACCCATAACTGTGTCCCTGGTGCG
CCTTCGGGAATACCAAGAGATGCCTGAGTGTTTTCGCTCTTACATCGTCGCTAAGGCCTCCCGGCGTTTCAATATTCGGTTCTTTGGGGCCGGTGAG

ATTGAGGGTTCTTTACAGGAGCAGGAGGCAACCGCCTGGGCTGAATGCCAGGAGTACGAACTTGATTATGGTAACTACAACATGCTCGATGGTGAC
GCATGGACTGGTGGCCGAATCTCCCGTTAATCCCAATAACAAGGAGGTCACTACATGGGTCTTGTTTCCCAAAGTGTTAAGAACCTTAAAGGAGGT
ATATCGCAACAGCCGGACATTCTGCGCTTCAGTAACCAAGGTAAGGTACAAATCAATGGATGGTCTTCAGAAACTGAAGGTCTACAGAAGCGCCCA
CCAACGACCTTTCTGAAGCGCTTAGGTGCTGTGGGTATGTTTGGCGCGAAGCCTCTTATCCACCTCGTGAACCGTGATGCATCCGAACAGTATTTTA
TGGTGTTCACGGGGCAGGGCGTTGAAGTTGCGGACCTCAAAGGGAACCGCTACACCGTCCGGGGCTACAACGGTTATGCCAACTGTGAGCATCCA
CGGAATGACCTGCGCTTAATCACCGTCGCTGACTACACGTTCGTTACCAACCGCAACAAGCTGACAGCAATGAAAAGCACGCTGACCCATGCCGA
CTACGGTTCTATGACGAGGCGCTGTGTAATCAACGTCAGAGGCGGTCAATACGGCCGAACCCTGAAGATACACATCAACGATGTCCAGATGGCAAT
CCTGAAGATGCCCGTAGGTGACTCTGTGGTGACTACGCCGCCCCAGGTGGAGCAGACGGATGCCGGTTACATCGCTACTCAGATGGCCGCACAGAT
CAACACTAACGGCGCTTCTCGCGGGATCACCGCACAGGCGGGCCAAGGTTGGATCATTGTGACAGCCGCTGGGAACGACATTAAGACCGTGAAGA
CCGAGGACGGCTACGCAAACCAACTGCTCAACTCGTTCATCTATCAGGTCCAGACGTTCGCCAAGTTGCCCGCTCAGTGCATTGACGGATACCTCG
TGGAAATCACAGGGGAGGCATCACGTACCGGGGATAACTATTGGGTGCGGTATGACTCAGCGGGGCAGGTTTGGAAGGAGACCGTTAAGCCAGGG
ATCATTACCGGGTTTGAGCCAACGTCTATGCCTCATGCCCTGGTCCGAGCGGCTGACGGTCAGTTTGACTGGAAAGCCTTAACGTGGCCTGACAGA
ACCTGTGGGGACGACTTAACGAACCCCATGCCCTCGTTTGTAGAGGCGACCATTAACGACATTTTCTTCTTCAGGAACCGCTTAGGTTTCCTCTCGG
GTGAAAACGTTGTGATGTCCAGGACCTCGAAGTATTTCAACTTCTTCCCGGCCTCTGTCAGCTCCATCAGTGACGACGATCCTATTGACGTAGCGAT
CAGTCACAACCGGGTGAGCATCCTAAAGTACGCCGTACCGTTCTCCGAACAGTTGCTGCTGTGGTCCGATCAGGCTCAGTTCGTTCTGTCCTCTCA
GGGGATCATGTCGACCAAGACGGCCCAGTTGGACCTAACCACAGAGTTCGATGTGAGCGATGGGGCACGGCCTTTTGGGATTGGGCGGGGTGTTT
ACTTCGCTGCACCGAGAGCGGCCTATACGAGCCTCAAGCGGTACTACGCGGTGCAGGATGTGAGCGACGTTAAGTCAGCAGAAGACATCTCTTCG
CACGTTCCAAGCTATCTGCAAAACGTGGTCTATCACATCCACGGGTCAGGTACTGAGAACTTCGTTTCCGTCCTTTCGGATTCCGAAGAAAACAAG
GTCTACATCTACAAGTTCCTCTACCTCAAGGAAGAGCTGGTACAGCAGTCTTGGTCACATTGGGAATTCGGTATTACCAACCGGGTCCTGGCGGCTG
ACTGTATTGGTTCGTATATGTACCTGCTGAATGAGCGCGCAGGTGTCGGGATGTGCCTGGAGCGCATCGAGTTCACAGCGGACACCGTGGACTACA
TGCAGGAGCCTTATAGGGCCTACATGGACATGAAGAAGCTGATGACACCTACGCGGTACGACGAGGACCTGAATGAGACCTATGCGAGCCTGAGT
GACATCTATGGCGGTGTTCCTGATCCCCTTTCAGTGTTCTACACCCTGGACCCACAGGGCGTCTTAGAGCGCCACACCTCGGACAACTGGAACGTG
GACGACCGGATTAAGTTAGTCGGGAATCGCATGGGCACGACTTTCATCGTTGGTCATGAGTACGGCTTTCAATACGAGTTCTCAAAGTTCCTCATCA
AGCAAACAGCAGATGACGGTACGACCAATACCGAGGACATCGGGCGTCTTCAGCTACGGCGAGCATGGGTGAACTACGACAATTCGGGCGCCTTT
GAAATCAACGTGAACAATGGGTCCACACAGTTCGTGTACGTAATGGCAGGTGGGCGCCTGGGCACAGAAGCAACTCTTGGCGGGCTCACGTTGGG
TACAGGGCAATTCAAGTTCCCAGTCACGGGCAACGCCAAGGCTCAAAGCGTAACCCTTTCGTCCTATGCACCAGTCCCTCTGAACATCATCGGATG
CGGTTGGGAGGGTAACTACATGCGCCGGTCAAGTGGCGTGTAAGGAGATAACAAATACAAGGAGGAATCTATGCGACTGATCAAGGCCACTGAGG
CCCACTTAAAGGCTGCTGCTGCTGACCTTTGCGCCTCGGACCTTCAAGAGTTTCACTGTATGAAAGCCGGTAGAGACCCCCGCGATGTTCTCCCGG
AAGCAATTGATGAACACGCCCAGGCAATCGTCGTGGGCTCCCTGGTCTTGGCAGTAGGCGGGCATACCGCGCTGGGTATCTGGTTCGTGACGACCA
ATGTGGTCCACATGCTGACCCCAGCCGAACGCTTCCAATTTTACCGCCTGCTTAAAGCGAACCTTGATCGGGTCAAGCAGCGCTCACCGAGTCCTA
AAACCAACTGGGTTTCTGTGGACAACCACGCACACATCCGCTTGCTGACAAAGCTGGGAGCCTCCTTCAACGAGGGCATCTTTATGTCACCTGCCG
GATTTGCCTTTAAACAATTCTGGCTATAGGAGGGCGGCGCTATGTGCGAACCCGTAAGCATCGGTCTGGCGGTTGTCGCCGTGGCTGGTGCAGCAA
TGAGTGCATCGGAAGGGGCCAAGGCTAAGGGCCAGATGACAGACAACGAGCGTAAGTCCCAAAACGAAATCGTGAAGCAAGCGAACTTCGCGG
AGAACGATCAGAAACTTGCAACCGTGGATAAACACGATGAGGCCCGCCGTCAGCTCACTGAGGTGAACCTACAGGCTCTACGGAACCAAGGGAC
CATCAACGCTGCTGTAGGCGAGTCGGGTCTTGCGGGCAACTCCATGGACCGCATCAAGAACTCTGTAGCCAACGATGCATCGGCGCAGAAGATGA
ACATCACCGAAAACTACGAGCGGGACTACCAGGCGATCTTTGCGAATCGCGTGGGTAGCTCTGAGAACTCTAAGGCTGCTGTACGTGGTGCAGGT
GGCAATAACTACAAGGTGAACAACATCGCCAACGCCCTGAATATCGTATCGGCTGGTGGCAGTGCCTATACGGCATCTGGTGGCAAGTTCGGTAAG
GGCGCTTCAACGACACCAGCGGGAGGTAAGACAGCGTAATGGCTAATGATATTGCACAAGCACTGGGCCAATCAGCCCAAACAACAGCGGGCCGT
CAAGGCTTCCGAGCGGGGTCTGGTACAGGCTATCAGGCTGCACGAATCGCTAAGCCCGTCTTGGACACCTCCCTTGGGGATGCCATGCGGAACTTC
GTGAAAGCTGGTGGGGATTCCTTTGCGTCCTACGAGCAGAACCGAAAGTCTCAGGCAGACGAGCGCTCAAACGAGATTATCCGAAAGCTGACCCC
TGAGCAGCGCCGACAGGCAATCGGGGAGGGGACTTTGCTCTATGCAGATGATCCAGACGCTATGCAGCAACTGCGCCTCAAGTCGGGCCGTAACG
CTGCCTACGAGGTGGATAACGAGGTCCAGACGGAAATCCAGAAGGGGTCTTTCCGATCCCGTAAGGAGCTGGATGAGTGGCGCCAAACTCGCATG
GAGCAGAAGTCCAAGAACTACGCTGAGATGGCAGGTATTGATCCTAATGACACCATGTACCAGCAAGGTTTCAATGCGGACATCACTCAGCGTAAC
GCTGCCGTCTACGACCTGCACTCACAGTACCTCTCCAAGAACCTTGAGACCCAAGCGGTACTTGAGACCCAAGCTGACCTTCAGCCTATGCTGGAT

GACCCGAAGATCCTGGGGAACCCTGCGAGTGCGGAACACTTTGCGAGCTACTTCAATAATAACCTCAAGACCGGCGTGTTCCCGAGTGACGGCCA
CGCGGTTCAGGCTCTTCAACGAGTGGCCTCAGCGGCTGTAGAGAAGGACGGCGGCGTGACGCTGCTGGATAACTTGGGAGACAAGACGATCAAC
GTCTACGGGGGCCAGCGGAAGGTCGCGGACTTGTTGGGTCCTGAGATGATGGACAACCTGAAGATGAAGGCCGCTAAGTCCACCTACACGCGCAA
CTCAAAGCGAAACGAAGAGTTCGAGCTTGGCCTTGCCAATGCCGTGCATCAAGTGGACCCAGCAGCAGGTTGGCAGGCTCTCAATAAACTTGAGC
AGGGGAACGATTGGATTCAGACGGGGGAGCAGATGACGCCTCAACGTCAAAAGCTGATCGACGCTAAGAAGCAGATGATTGCCAAGGTGGCCCA
GGACTCACAGGCTGGTCTCTTGAAGTTGCAACAAGCTGCCCAGGCGGACAACCGACAGGCTGTAATCGAATACGCTTACGCGCGGCGAATGAGTC
AGGAGAACGTCGCTGTATCCCCTAAGTTCCTCCCAGTGAATGACAGTACCGGGGAGTTCAAAGAGAGTGACATGGCGACCTATGCAGCCAACAAG
CTGAATCAGATCAACGCCATGTCCATCCCTGAAGAGAACAAGTCCGCAATGAAGGCTGCATTGCTTCGGGCTGACTACGACAAGGGGCCGTTTCA
AGAGAACTTCAAGACCCTCTTAACGGACGCCTCTCGGGAGTGGCAGGGCGCTTTGCTCAACCCTGAGCCGACTGCGGACATGCCTCGCCTGAAGG
AGCTGCAAGCTGCCTATCAGCAGGACCCTTCAACCATTGCTCAGCTCTTCCCGGATCAAGCGGGCCTCCTTGAGAAACTTCGGTATTCGGCAGAGT
CGGGCATCGATCCACAGATTCTGATCGACGCTGAGAAGTCCAAAAAGGGCCTCAGCAAGGACGAATCGAAATTTAGGGATGAGCAGTGGTCTTCC
GTCAAGAACGACAGCGGATACAAGGAACTGAAGTTCATCCCTGGACAATTCGAGAGTCAAGCCCGTCAGGTATTCGATGCGGTGACATTGCGGTC
TGGTGATTCCAACGAAGCAGCAAGTGCCGTCTCGGAGTACCTGAAGAAAACCGCTGTGTCATTCACCGATGACAAGGGCACCTTCAGCTCTAACA
GCTTCCACGGGATGGTCAGTAAACGGGACCTCATGACGGACGTAAACGATGTCACTTCATGGGAGACCGGCAAGTCGATCATCGACGAGACCATG
CAGAACCTGCGTAAAGACGCCGACTGGGGCGCGGGTGGCCTAACCGTCACTGCAACCCAAGGCAACATCATCATCCAGTCCCTTACGGGGCGACG
ACTCCGACTCAGTAAGGATGCCCTCGGGAACCTTGCGCGGGATCGTGCTGCACAGCAGAAACAAGCGGCCTTCGACAAGAACGTGAAGTCTGCG
CAGAAAGATCAAGAGCTACACGACCGGTACATCCTCGGAGGTGCCCGCAAACAATGACCTGAATAAAGGAGACCTCTATGAGTAACGACTATGAA
TCTGTGAAGGCCAAGGGGACACCCTACGACGCCATGATTCGACAAGAGGCTGACCGTAATGAAATCCCATACGAGTTCATGCACAAGCTCATCTAC
AACGAGTCGAGCTTTAACAACAACGCCAAGTCCCCTACAGGCCCAAGGGGCCTCGGACAGTTCACAGAGTTGACCGGTAAGGCTTACGGCCTGAT
GACCCCTGAAGATCGTATGGACCCACAGAAGGCAGTCCCTGCGATTGCCCGCCACCTCAAGGACCTCAAAGGCGCCTATCAGGGTGACTTGCTGA
AGGCCGCTTTGGCATACAACCAAGGACAGGGCCGTTTGGGCTCCCCTCAGTTGGCCGCACTGGATCAAGGTGACTTCACCAAGATCAGTCCCGAG
GGTCAGAACTACATGCGCAAGCTGCTGGACGTGTCCGGTGATTCGCCTTCCCGCCAATGGTTCGATGCCCAAGGAGTGACCAATCCCGGTATCAAC
CCAAAGGCCCAAGCCGTCAGCTTTGAGGATGCTACTCGGGGTGTGACGGCAGGCTCTAAGGTTCGTGTGGGTCAGGACCTCCCTCAGTTGGGCAA
TATGAATCTGGAAGGGGGCACAGCCCCTCAGACTCAACAAGACTTCATCACCCTTGAGAACCACATTGGGAAGCCTGCAAAGGGCACCTTCGAGG
GTACAGGCGATGCCACAGCAGCATCTCTGGCTACCTCACCGATGGCCTCTCTGTTCCGGTACGCGACACAGGAAGACCATGACCCGCTGGACTGG
GTGAACCCAAGCGATACCTCTCAGTGGACTGCTGACGATTACGACATGATCCGCAAGGAGGGTGTGGACCCTCAATACTTCAGCTTCGTGCAGGAC
TACGCCAAGGGCAAAAGGGCCAACCTTCCGAACGCCATTGCGATGGCAAAGGAGAACATGGCCTATGAGCAGCGCATCGGTAACGCTGGGACAG
GCGCTCAGATTGTCGGTGGGTTTGCGGGGGCCGGTCTGGACCCTCTGACCTACACGCCTATTCCAGGGACAACCGGTGTGCGCCTCATCTCTCGGG
TTGCTCAAGGTGCGATGTACTCGGGGGCGGCTGCTGTTGCCTCTGAGGGGCTACGTGAAAACATCACGGGCATTGAGGGGCACTACGGGACAGCT
CTCGTGGGTGGTGCATTGTTCGGTGCCGGGGCTACAGCTCTGTTTGATAAGGCGCTGAGGGCTGCACCGCATGAGGCTCCCCGCGTAGATGTTCCA
GACGCTGACCTTGAATCGATCCTTGCGCGACACGGTGAGTCCGCCTTGCCGAATGAGCGCTTTGACCTGCATCCCGATATGGAGCCTGACTTGAAC
GACTTCGCGGGTCCGAGCATTCGCTTGGAGGCCCGTGAGACCGCTCGCCAGTTGGGTCAGGATGATCCTTCACGGTTCCCATGGTTGGCCGGTGAG
GAACCCAAGGAAGCCTTTGGGGTGAGCTATGTTGACCATCCTAACGAGCCTGGGGCCGTGCGCTTGCAGGACGGTTCAATCCTGTCTGCTGACAAT
CCATTGAACCCTAAGACCCTTCAGGACGCCATGGAGGTTATGCCGGATGACCGTGCAGCCCGAGGGGTGACTATGGGCGGCTTTACCGAGATTGGC
TACACGCTGAATCGCTCGGAGAACTCTGAGATTCTGGCGATTGGTAATCAACTGTTCAGGTCTCCTACAGGGACCATCAGCGGCTCTAACGGGAAG
TTCGGGGCTACCGCCTCGGACATCATTGAGCGTATCCGGGGCCAGGACCATGTGACCTACAACAAGATGTCTGATGAAGTTCTTGAGGCGATCAAA
GACCCAAGCTATGCGATGACTGACGGCGGTCGGCAAGCTCACATTGAGCGTGCTTTCAGGCGTGTCGCTGAGGCCACTGAGGACCTTTCGGGTTC
CAAGGCGGCGCAGCTCACAGCCGGTGAGAAGAAGCTGATGGGCACCGTTAAGGCTCACTACGACCGTAAGGCTGAATACCTTGCAGCGCCTGCCC
AGTTCGGCAACAGGAACGCCCGAGCGGTTCTTTCGGGCACCCGTCACGCTGGCTCGTACATCCCGAATGTCTACAGCTCAGCCGCGAAGAACCTG
AACATCCAACGCTTCGGTGGGGCGGATGGTCTGCGCGATGCCATTAAGGAGTCGTGGTTGTCTTCCTACGCTGCACGGCCTCACGTAAAGACTCGG
GTGGACACTCATCTGAAGGCGCTTGCGATTGCCGAGGGGCGCGTACTGGAAGACAAGGACCTTCCCGCCGCTGTTGAACTGTATGCCAGCAACAA
GGCATACGGCATTAGCCACTCGGACCAGTTCGACCGTGCAACGCTCATCGACAATAGTGCCGAAGGTCTGGTCGGCGTGGAGAACAACAATTTCCT
CGAAGGCCGTCACCTGTTCGATTCGGACATGGCGGTGCCCCTTAGTGATGGCTCACAGTTCTCTGTGAATGACCTGCGTGAGTTTGACCTGGCGCG
CATCACCCCAAGCTATGACCGCCGTGTGAACGGTGACATTGGGATCATGGGGGCCACGGGGAAAACTACGAAGGAACTCAAGGCTCAGATTGCAG

GGGTCCAGAAGGGGAAGGGAACGTCGCAGGAAGTGAAGGCATTGCAGGATGCGCTCAAGATTCTGACAGGCCGTGCCCGAAGAGACCCCGAAG
GCGCCCTTGCAACCGCTTCCCGTGCCTTGGCAGACATGAGCTTCTTCGCCAAGAACGCCTACATGGGCGTGCAGTCAATAACTGAGGTTGCCGCAA
TGGTGACTAAAGGGCACACACGGATGCTCCTGAAGGGCGTCCCACTGCTCAAGGAGATGACCGCCTGGGGCTCCAAGATCAAGGCAAAGGACCT
TGCAGACATGCACTCTATGGTCTTCGGTCGGGAGCTGGATAACCACATTCGGCCAACCCGCCAGGACATCATCCAGCGTCTCCGAGAGCAGGCAGA
AACAAACCCTATGCTTGCCCAGGTTGTGGGCTCTGTGAAGTACACCACACAGGAGCTGTCAGCTCGATCACCGTTCACCAAGTTCCTCACAGAGTC
GTCTAACTACATCGCGGATGCAGGGCGTTCCGGGGTCCTCTCGGACTTCGTGAATCATGTGGTGGACGGTCGGGCCTCGAAGCTCTTTGAGGACAC
CCGCCTGAAGTCTATGTCTATCACCCCCGAGCAGTTCGCGGGAATGAAAGACCTGATTAAGACCTACGTGAAGAAGCAGCCTGACGGGTCCTACA
AGATCACCGATAGAGCAGCCTTTCAGCGTGACCCTCGGACAATGGACCTGTGGCGTATGGGCGACAAGATTGCAGATGAGACGATCCTACGACCA
CACAAGCTGTCTTCGGTAGACACCGAGGCATACGGTGCTGGCGTCAAGATGGCGATGCAGTTCAAGAACTTCACCATGCGTTCAGTCAACTCCCG
GCTCATCCGTGGCTACCACGATGCCACGAAGAACGGTCGTGCAATCGACCAGACCATGCAGGCGGTTCTCTCTACGGGACTGGCCGTTTCTGGCTA
CGTAGCGATGAAGTACAGCCAAGCGGCGGGGATGCCAAAAGACCAGCGTGAGAAGTTCCTACAGCAGTCCCTTGACCCCAAGATGCTTGCCTATG
CGGCTCTGTCGCGCAGCTCCCACATTGGGGCTCCTTTGGGGCTGGCGAACATCGTAGCGGCACCTTTGGGATTTGATCAGGCCGCTGCTGTGCGCT
CTTCGATTCTCCCACGTGGTCCCAAAGAACAACGCCCTCAAGGTGCCATGAAGTACTCCCCCACGAAGTCTGAAGGGTTCACTGGGTTCCTTGGCC
GGGTATCCGAACAGGTTCCAGCAATTGGAACTGTAGCAAGCGGGTATCAGGTGGGTTTCAACTCAATGGGGGCTGCTGGTACTGACTCCCGTCGAG
CTGATCAGGAATACATGACAGGTGTCTTCAACGGTCTTCGCGGCCTCGTGCCGAATGATCCGGTATCGCAAAAGCTCTTGCTCATGCTGATGGAAG
GGCAGGGCGTAGAGATTCGTTAAGAAAGCGCCTCCCGCAAAAGCCCCCACTATAAGCAGATGTCACCACGAGTCTGCTTTCTCATTCATAACTCAA
GGAGGACTTAATGGCCGCTCCCAAGACAGTCCTAACGTATCCACTAAATGGTGCGCAAAAGGACTTTATAATTCCATTCGAGTATTTGGCTCGTAAG
TTCATCGCCGTGACCCTCATTGGTAAAACACGGCAGACCCTCACGCTTAACTCCGACTATCGGTTTACTTCCCGTACCAATGTCACGACCACAAAGG
CGTGGGGGCCGGGGGATCAATTCACAAGTATAGAGATTCGCCGGGTTACTTCGGCAACTGATCGCCTCGTTGATTTTGCCGATGGGTCGATTCTTCG
GGCCTACGATTTGAACACCTCACAGGTTCAATCGCTGCACATTGCTGAAGAGGCCCGTGACCTGACGGCGGACACCATTGCGGTGAACAATGATG
GTGATTTGGATGCCCGTGGTCGGCGCCTTGTTAACCTTGCAGATGCGGTGTTGGATGGTGACGCCGTTACCTTGCGGCAAGACAAAGCATGGGGCG
CCTCGGCGTTGAATCAAGCTAACCGCTCTGAGCAACAGGCAAACATTGCGACCCAGCAAGCTAACATTGCGGGCTCTCAGGCGCGAACTGCGGTT
GACGCGGCGGGAACATCCACGGTCATGGCTAATGAGTCGGCGCGGCATTCTGGTAACTCAAGCACATCGGCGGCACAGTCCAAGGAATCCGCTAC
CACTTCGATCGCCAACTCGAACCTTTCGAAGGCATGGGCTAACGCCGCTGAGGATGTTCTGGTGACCCCCGGCCTGTACTCGTCGCTGCACTACTC
CCGCAAGTCCGCCAATAGCGCGGCTCAGGCGTATCAGGATGCAAACCGTTCGACCACTCAAGCTGATCGATCTAAGACCGAGGCTGACCGTGCGA
AAGTTGAGGCTGACAAGCTGGGTAACGCGAATGCCTTCATGGGCACTATCTTCGATGTTCAGGGTTACGCTGTACGCTTCCGGGGTACGCATCTGAT
AAAACAGGCGCTCTACATTCAGAGGCAGGGCACAGATGACATAAGTGACTTTGTTAGCTGGAGTTACGAGCCTAACGGTGCTGTTTATCAGCGAAG
TCGTTATGGGGTGCCGTTCACGAGTCTTGATCCAAGCGGCAACTTTGCTGTATCCGGCACCATCACCGCAAACTCTGGCTCGTTCTTCTCTGTGTCT
CCTGCTGACTCGGGGAACGCTCACTTATGGTTTGACGGTCCGAGCGGGAATCGCGGAGTAATCTACGCGGGCATTGATCAGGTAATCCGGCTCCGC
GCCGGTCGAACCACAGTGGGTATGACCGTGGAGCCTAACGGCAAGGTCAACACTGCCACCGATCTTATCGTGGGGGGCTCTATTGGCCTGCCTCAA
GCTACCATCCCGCCTAACGCGGACATCCGCGCCGAATGGATGACTGGAGGAAGTCTTTATGGAGCGCTTAGTCGCGTCACTCCTGGCACGCGCCTG
CGTAACACCCCCTCGGTAATATGGGAGCGTGGTGTGGACACAGTTGGAGGTGTAGGTGACCAAGGGGCGACGGGTTCTCAGTTTTATCTCCGCGA
GCCTTTACAGTCCGGTGACTTCTACATGTTCGAGATTTACGGCGGCGGCAAGTTTTATTCGACAACCAACTGTATTCCTTTTGGTGAGTCTGCAACC
GAAGAACACAACGTGCAGTTTGATAGCGGTGGGGCAATGCTGTTTACCCTTACTGAAGGCGGGCGGCGCATCACTGTGAAGGCTGGCAAACCCAA
TAATCAGTATGGGATTCGGCGTGTGTTCATCTTCAAACTTACCATTGCCTAAAGGAGTGCTTAATGAGTCTTGAAATTGATTTACACAAGGGGTGGT
TCGCGCCACTCCTGTTGTAGCTGCGGCTGGTGCTGATGTAGCAAGCCGTTTTGCTGGTTTAACAATGTCCGACTGGTTCTATGCAGCGGCCATTATC
TATTCGCTGACTCAATGCGCCGTCTTGGTCTACAAGACTGTGAAGGGGGTCCAAACAAAACAGGAGGGTTAATGTCGCAATCTATTCTTGAACAAC
TACTTGAGGCAATCGACACAGAGACAGGTAAGAATCTTTTGCGTGACCTACAGAAGCCTGAGCTACGAACACCTCAGCTATACAACGCCATCGGCA
AGTATCTCGACCGTCACAAGTTCACTATCGCTAAGTTGCAACCTGATGAGTCCCTACTGGGTGACTTGGCGTCTGCGCTTGCGGAAGTCCCTGAGTT
GACCGACGAAGAACTTTATGGTCCAGCAACTCAACACTAAGGAGCCACAACCATGACAACCCTTACCAAGGTTCTCTTGGGGCTTCTGTGTGCTTG
TCTGATAGCCCTTGGGATTTACTTCAAAGGCTATCACGACTCTGACCAGAAACACCTTAAAGAGGACGCTGAGACAGCTTTAGTCTACAGGCAGAA
ACAGGAGGAAGAACGTGAACTATCTCAAAAGGCTTTGGCGGATATTTCTTTGCAATGGCAAGCGTACCTTAAAGACCAGTCGGCTTTGGCTGACGG
GGTTATTGGTAGGTTGCGTAATGACGGTATCAGCCTGCGGGTCAAACTTGCCGATGCAACCGTGCGTTCAGTCACAAGTGCAGGTAGACCCGTCCC
TGATGGTTACGCCGACTTATCAGAAGACACTTCTCGATTCCTTATCGAGCAAGCTCAACGATGTGACGCCCAAGTAACGGGCCTGCAAGGAGTAAT

TAAAGAACTGCAAAGGAGTAAATGAATGATCATTAATTTGAGGGAGCACCACGACAAGAAAAAAGAACAAGCTAAATATGATGCAGAACAAGCCG
CACAGGAAAAGATTCGGGATCGCGCTAAACAGGTGAAGTGGCCGGGTGATCCTGAGTGACCCGAACTGTTCGCACTGGCGAGCAGGACATAGCG
TTAATGAAAAGGTCCTTTGTGGCCTTTTTGTTTGTCCTGTGGCGCGCTCTAAACCTCCCTAAGCCAACCAAGTGCCAGATCGACATGGCACGTACCC
TAAGTGACGGAAAGCAGCGAAGGTTCATCCTGCAAGCCTTCCGTGGCATCGGCAAGTCCTTCATTACGTGCGCCTTTGTGGTCTGGAAGTTGTGGA
ATAACCCACAGCTCAAGTTTCTCATCGTGTCGGCCTCGAAGGAGCGGGCAGACGCCAACTCCATTTTCATCAAGAGAATCATCGACCTACTTCCGT
TCCTTTCAGAACTGAAGACGGGGCCAGGGCAACGGGATAGCTCGTTGAGTTTCGATGTGGGACCGGCCAAGCCGGACCACTCGCCCTCTGTGAAG
TCTGTGGGTATCACCGGCCAACTGGCGGGCTCCCGTGCTGACATCCTTATCGCGGATGACGTTGAGGTTCCGAACAACTCCGCTACGCAGTCCGCA
AGGGATCACCTGGGCGAACTCGTGAAGGAATTCGATGCGATCCTGAAGCCGGGTGAAGCATCCACCATCATCTACTTGGGCACGCCCCAAACTGA
GATGACTCTTTATCGTGAGCTGGAAAACCGTGGCTATGTGACCACCATCTGGCCTGCGAGATACCCGAAAGACCAGAAAGACCGTGAGAGCTACG
GCCATCGTTTGGCACCAATGATTGCGAACGAGCTGGAAGCTGACGGACCCTTTACTGGAAACCTACAGACCCTGTTCGATTCGATGACACTGACT
TGCGTGAGCGTGAGCTGTCATACGGAAAGGGCGGCTTCGCTCTTCAGTTCATGCTTAACCCGAACCTGAGCGATGCCGAGAAGTACCCGCTGAAA
CTGCGTGACTTCATCGTGGGGCGCTTCAGCCTCGATACAGCCCCTACGACCTTGCAGTGGTTGCCGAACGGTAACAACGAAGCCAAGGGCCTGCC
TAACGTTGGCCTGAAGGGTGATCGTTTCCACCGCTACGAGACTGTAGGTCAAGCGTCTGCACCTTACTCCGCGAAGATCCTTGTGATCGACCCGAG
TGGTCGCGGTAAGGATGAGACAGGCTATGCCGTCCTCTACATGCTCAACGGCTACATCTTCCTGATGGACTGGGGCGGGTTCCGTGGTGGCTACGA
TGACAAGACCTTGCAGGCCCTGGCAGACATCGGCAAGCGCTTCAAGGTCAACGAAGTGATCATTGAGGGTAACTTCGGTGATGGTATGTACGTGA
AGCTGTTCAGTCCTGTGATGACCCGTACACACCGTTGTGCAGTCACTGAGGTCAAGTCAAAGGGCCAGAAAGAGCTACGCATCTGTGACGTGCTG
GAGCCTGTACTGGGCAGCCACAAGCTGGTGGTCTCCGAGGATGTCATTACGCGGGACTACGAGACTGCGCTGAACATCGACGGTACGACTGACAT
CAAGTACAGCGGCTTCTATCAGCTCTCAAGGATCACCAAGGACCGGGGCTCACTTGCCCATGATGACCGACTCGATGCCCTGGCAATCGGCGTTCA
GTTCTTCACAGAGGCCATGGAAAAGGATTCTGAGGTGGGAGCCTCGGAGATGCTGGAAGAGTACCTAATGCATCACATGGAAGACCCGCTTGTGG
GCTTCGAGGATCACCGGCAGATGATGATTGGTGACGTGATGATGCAGTACGAAACAGACGGAACGACCACCAATTACATGGGGTGGTGACTACGTA
AAGGGGCCTTCGGGTCCCTTATTTTTCAGTTTGATCAGGGCTACAAGGCACGTCCTGCAAGGGCTCCAAAAGCACCCACTATAACCATAGGGAGGC
TTAAAGTTACTCTCTTAGAGATGCCTCTGAGACCTCCTGTGTGGGTGGTGGTTATCACCATCTACCACCTTCTCTATTACCAGCTCAAAGGAGGCTG
AAGCATGATCGTTATGACTACTCGCTGGCAGACCTTTGTGGCTGTCGTTAAGGCTCTGGCGAAGCACCGGGCTACGTACAAATTCCTTGCGCTGCTC
TTAGTGACTCTCGGAGTTGTTCAAGGTGGTCCACTTGTCGAAACCTTTGGGGATGTCGTATGTGTTCTACTTGGTGGCTGTGACGGGTGATCTATGC
GGGATTCTCGTAGGGCTATTAGGGTGAGCCGTGGTGAAGCGACTTAACGAAGAGGGTTATTTTTTCCACTACATGTACTCGGTCGACTTTGAGGCCG
CGCTTGATTCAATCAAACTCATCAAGCGCTACAAGAGGCCGGACGTCAAATTTGCATTGCTGCGTGATGTCACCGTGGCTTACATACGGCCCTTCTC
AAAGAACAGAGGAAGGAAGGCCAAACATGAGCTTGGTGTTAATGATGTTGTCCCCAAGCACCTTCGAGACCTTCACGATGTGCTTTATAAGCTGC
GAAGCGAGCAGTTCGCCCATACTGATCTTACCTATTACGACCCGAAGTCAGTCATATATGGAGAGGGCCCTACGAGCTTGGTCTACAACGGGTTCAG
CTATGAGGGCCTATTGGCACGCCTTCCAGAAATAGAAGAGCTTGTGAAAGCCGTAGAGAGCGTGGTGATTGCCCGCATCAGGGCATACGAAGCAG
AGGGCTCTGACCTCAATCACGAATCAGCGATTAGGCTTGATCCAGCCCTATCGAAGCTGTGGGGGCTCCTTTGAGAATCTGACAGAAAAATCTGAA
GGACCACCTCAACTGTTCTATGCCGCCAGTTCCCCCCGTGGCCCCTCTCGCGCCTGAGCCTTATCGCATGGGTGCGCCTGGGGTCCTTATCACGGGC
CTGCACGCCTCTGTAAGTCACCCATTCCACTGTCACAAATCGTTAGAACTCTGTTAGACATACTGTTAGAACTGGTTGGACAAGCTCTCTGAGATAC
CCATAGAATGGGCCTTCCAGCCCTCATTGGTGGCCTTGC
